# Identification of Novel Non-secosteroidal Vitamin D Receptor Agonists with Potent Cardioprotective Effects and ﻿﻿devoid of Hypercalcemia

**DOI:** 10.1038/s41598-017-08670-y

**Published:** 2017-08-16

**Authors:** Santosh A. Khedkar, Mohammed A. Samad, Sangita Choudhury, Ji Yoo Lee, Dongsheng Zhang, Ravi I. Thadhani, S. Ananth Karumanchi, Alan C. Rigby, Peter M. Kang

**Affiliations:** 1 Division of Molecular & Vascular Medicine, Beth Israel Deaconess Medical Center, Boston, MA 02215 USA; 2 Cardiovascular Division, and Beth Israel Deaconess Medical Center, Boston, MA 02215 USA; 3 Division of Nephrology, Beth Israel Deaconess Medical Center, Boston, MA 02215 USA; 4 Renal Division, Massachusetts General Hospital, Boston, MA 02215 USA; 5 ChemBio Discovery, Inc., Lexington, MA 02421 USA; 6 Warp Drive Bio, Inc. 400 Technology Square, Cambridge, MA 02139 USA

## Abstract

Vitamin D regulates many biological processes, but its clinical utility is limited by its hypercalcemic effect. Using a virtual screening platform to search novel chemical probes that activate the vitamin D signaling, we report discovery of novel non-steroidal small-molecule compounds that activate the vitamin D receptor (VDR), but are devoid of hypercalcemia. A lead compound (known as VDR 4-1) demonstrated potent transcriptional activities in a VDR reporter gene assay, and significantly ameliorated cardiac hypertrophy in cell culture studies and in animal models. VDR 4-1 also effectively suppressed secondary hyperparathyroidism in 1α-hydroxylase knockout mice. In contrast to 1α,25-dihydroxyvitamin D_3_ (1,25-D_3_ or calcitriol), a naturally occurring VDR agonist, VDR 4-1 therapy even at high doses did not induce hypercalcemia. These findings were accompanied by a lack of upregulation of calcium transport genes in kidney and in the gut providing a mechanism for the lack of hypercalcemia. Furthermore, VDR 4-1 therapy significantly suppressed cardiac hypertrophy and progression to heart failure in both vitamin D deficient and normal mice without inducing significant hypercalcemia. In conclusion, we have identified a unique VDR agonist compound with beneficial effects in mouse models of hyperparathyroidism and heart failure without inducing significant hypercalcemia.

## Introduction

Vitamin D is a multifunctional, steroid hormone responsible for regulating many biological processes, including cell differentiation, cell proliferation, and the immune system, in addition to its more classic function in mineral metabolism^1^. There are mounting recent evidences suggesting that vitamin D deficiency may be a contributing factor in number of diseases, such as inflammatory diseases, cardiovascular diseases and abnormal bone metabolism^2–5^. Vitamin D signaling occurs when an active hydroxylated metabolite of vitamin D_3_ (1α,25-dihydroxyvitamin D_3_; 1,25-D_3_; Calcitriol™), binds the ligand binding domain (LBD) of the vitamin D receptor (VDR), a member of the nuclear hormone receptor superfamily of ligand-dependent transcription factors. This binding facilitates a series of conformational perturbations leading to DNA binding and transcriptional activation^6–8^. Despite the significant potential for vitamin D therapy, its clinical utility has thus far been limited by the fact that vitamin D also elevates serum calcium (Ca^2+^); hence the adoption of this therapeutic regime has remained controversial^9^. The high plasticity of the VDR signaling allows for tissue and cell selective VDR responses. For instance, VDR in renal cells responds differently than that in duodenal tissues^10^. Therefore it is attainable to develop selective VDR agonists with a high degree of cell-tissue specificity to reduce the hypercalcemic effect through chemical modifications^11^.

Congestive heart failure (CHF) is one of the most common health problems affecting approximately 5.7 million people in the United States^12^. Despite significant advances made in the treatment of heart failure, one of the most ominous statistics is that about half of people who suffer from heart failure die within 5 years of diagnosis^12^. Thus, much work is still needed in understanding and treatment of heart failure. Evidences suggest that alterations in the vitamin D axis are commonly associated with pathophysiology of the heart. Our laboratory and others have shown that vitamin D therapy blocks the development of cardiac hypertrophy and cardiac dysfunction in various animal models^13–16^. Clinically, there is strong association between vitamin D deficiency and the development of cardiac hypertrophy^17, 18^. In fact, improved survival rates and the cardiovascular mortality were found in hemodialysis patients treated with paricalcitol, an active vitamin D analog^19, 20^. These data suggest that vitamin D deficiency may be a contributing factor in the pathogenesis of CHF, and that the anti-hypertrophic properties of vitamin D signaling may confer a cardioprotective advantage.

Despite the significant potential for vitamin D therapy in cardiac hypertrophy and heart failure, its clinical utility has been limited by the fact that vitamin D also elevates serum Ca^2+^. Several groups have previously synthesized structural analogs that retain the selectivity profile of 1,25-D_3_, but are devoid of the calcification^21, 22^. Although number of 1,25-D_3_ analogues have been synthesized, few are of clinical interest at this time. In this study, we report the discovery and biological evaluation of a novel, non-steroidal compound that activates VDR, but does not trigger hypercalcemia in animal models of cardiac hypertrophy.

## Results

### *In silico* screens of molecular libraries identified agonists of VDR activity

**Figure 1:**
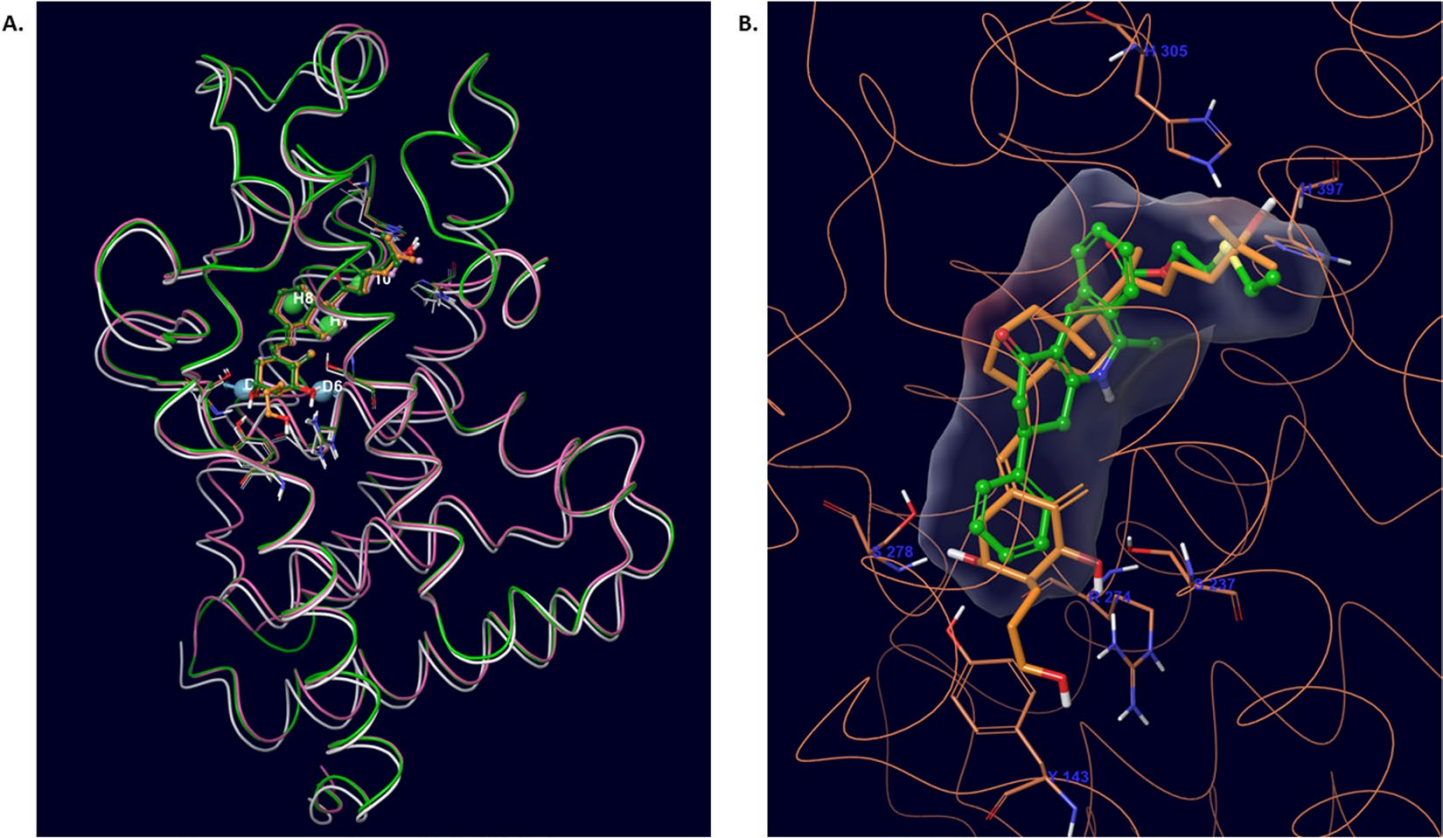
*In silico* screens of novel VDR agonists. (**A**) Overlay of three co-crystallized structures of vitamin D receptor (PDB codes: 1DB1, gray; 2HB7, green; 3CS6, pink), represented as thin tubes, their co-crystal structure represented as ball and stick, and pharmacophore (DDHHH.30) hypothesis (three hydrophobes, H7, H8 and H10 shown as green balls; two donors, D4 and D6 shown as blue balls with vector), depicting their potential interactions with binding site residues; (**B**) X-ray pose of co-crystal ligand in 2HB7 structure (orange, thick stick) and early lead compound VDR 4 (green, ball and stick) as aligned during pharmacophore screen (LBVS). According to ePharmacophore, the energetically important sites are H7 and H8 located in seco-steroidal fused C-D rings (corresponding to bicyclic ring in VDR 4), and two donor sites located on A-ring hydroxyls of 1,25-D_3_.

In order to identify novel non-steroidal scaffolds that bind to and activate the VDR, we used a ligand-based pharmacophore approach to bias the screening towards the critical binding features by extracting properties of the known agonists in pharmacophore query. This was followed by an *ensemble* structure-based evaluation of pharmacophore hits using an induced fit/ensemble docking workflow created to account for receptor flexibility. First, based on Glide SP docking screen in a X-ray structure co-crystalized with vitamin D_3_ (PDB code: 1DB1), we tested 35 compounds in reporter gene assay. We did not validate any of the compounds as agonist hit; instead we found 5 potent antagonists (Suppl. Table S1, compounds 1–5). In our second attempt, we employed a 5-feature pharmacophore query (Fig. 1) created by incorporating the common features of highly potent seco-steroidal VDR agonists, sketched and minimized within the binding pocket of VDR (1DB1 structure), as primary screening filter. This was followed by docking of 48,000 pharmacophore hits with acceptable (≥1.5) fitness scores into the 1DB1 crystal structure. This screen identified 8 weak agonists (Suppl. Table S2, compounds 1–8) and 5 antagonists (Suppl. Table S1, compounds 6–10).

**Figure 2:**
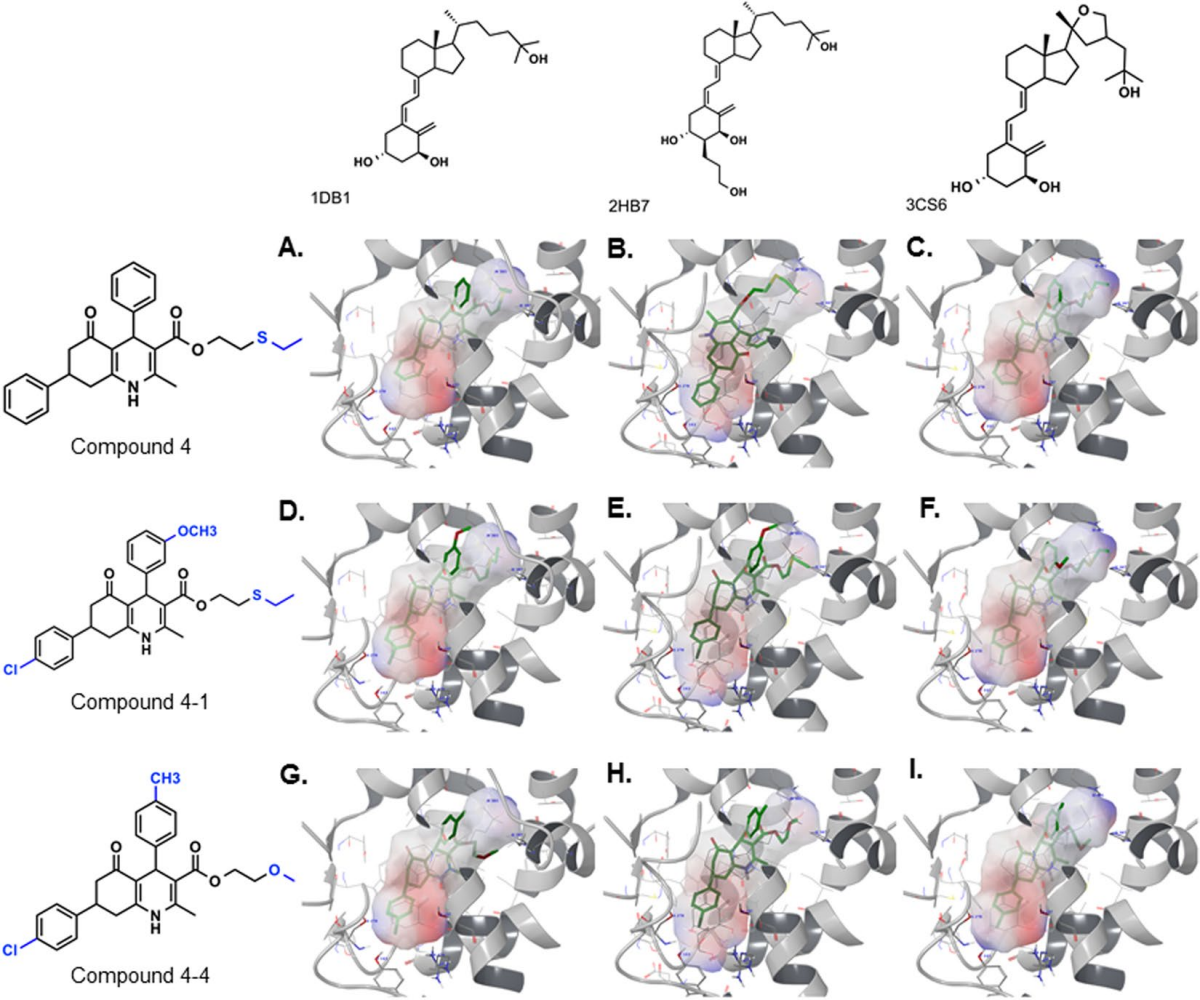
Binding of lead compounds VDR 4, VDR 4-1, and VDR 4-4. The long hydroxypropyl side chain on A-ring of 2HB7-ligand induces A-ring subpocket, whereas cyclized side chain on D-ring of 3CS6-ligand locally alter conformation of amino acid side chains. Binding poses (green, thick sticks) of VDR 4 (upper panel), VDR 4-1 (middle panel) and VDR 4-4 (lower panel) as predicted by Glide XP docking in 1DB1 (upper panel), 2HB7 (middle panel), and 3CS6 (lower panel) crystal structures. The space occupied by co-crystallized ligand in respective structures is represented as semi-transparent electrostatic surface (ESP) for relative binding comparison along with respective co-crystallized ligand shown in black wire bond representation seen within ESP surface. The residues within 3 Å distance of ligand are displayed in black wire bond representation, while secondary ribbon structure (shown in gray) is shown for residues within 10 Å of ligand, the 3 residues of helix (aa. 231–233) passing across the pocket are un-displayed for better pocket visualization.

Progressively learning from our earlier two screens, we then incorporated an *ensemble* high-throughput docking (HTD) approach to our partially successful virtual screening protocol. Given the conformational differences observed for several VDR-LBDs, we decided to use an *ensemble* of VDR structures (PDB codes: 1DB1, 2HB7 and 3CS6), to replace earlier single-structure protocol, to account for potential ligand-induced receptor flexibility. This *ensemble* based screening in three receptor conformations was performed in two rounds of ensemble HTD. In the first round, 48,000 pharmacophore hits were evaluated with relatively less exhaustive but fast Glide SP docking screen in each of the three VDR-LBD structures. In the second round, hits with SP scores better than –8.0 kcal/mol in each VDR conformation (~20,000 hits) were subjected to thorough evaluation using an extra precision (XP) scoring metrics available in Glide docking, which is documented to reduce number of false positives in virtual screening experiments, thereby improving the enrichment. Top scored 1000 molecules in each of the three VDR structures were analyzed for *consensus* rank ordering based on the four-score metric (3 XP docking scores from 3 *ensemble* structures and 1 pharmacophore fitness score). We selected 51 compounds to test this *ensemble-*HTD hypothesis in reporter gene assay that resulted in discovery of 5 potent agonists (Suppl. Table S3; compounds 1–5) and 5 weak antagonists (Suppl. Table S1, compounds 11–15). Compound 4 (Fig. 2; Suppl. Table S3; referred as VDR 4 hereafter) was selected as lead candidate for further chemical space expansion and potency optimization. The selection of VDR 4 analogs, obtained by substructure searches of known compound databases, was based on XP scores from ensemble-HTD and docking pose analysis, so that common scaffold retained the binding pose of VDR 4 and R-substitutions expanded the chemical space of A and D rings (R_2_–R_6_) of VDR 4 and R1 side chain (Suppl. Table S4).

### Reporter gene assay validated the activity of *in silico* hit compounds

To determine which compounds identified by virtual screens are actually capable of regulating the transcriptional profile of VDR, we performed a reporter gene assay using the GeneBLAzer® Cell-Based VDR assay. Reporter gene assay of 58 structural analogs of lead compounds VDR 4 and 5 (Suppl. Tables S4 and S5) validated agonist activity of 44 compounds (Suppl. Table S4; compounds 4-1 to 4–41 and Table S5; compounds 5-1 to 5-3) exhibiting similar or better potency than the parent leads. Six compounds (Suppl. Table S4; compounds 4–42 to 4–43 and Suppl. Table S5; compounds 5-4 to 5–7) exhibited antagonist behavior at 5 and 50 µM in this reporter gene assay. Based on agonist activity and predicted pharmacokinetic properties, we selected three lead compounds, VDR 4, 4-1, and 4-4 (Fig. 2; Suppl. Table S4) for further evaluation of their cardioprotective and calcemic properties in *in vitro* cardiomyocyte culture studies and *in vivo* animal models. The chemical structures of compounds were confirmed by NMR and mass spectroscopy, which showed overall purity of >99% by HPLC (C18 column and acetonitrile-water as eluent system) and as racemic mixture of diastereomers (retention time = 14.9 and 15.8 minute, respectively, for VDR 4-1 (Suppl. Fig. S1).

### Anti-apoptotic and anti-hypertrophic effects

**Figure 3:**
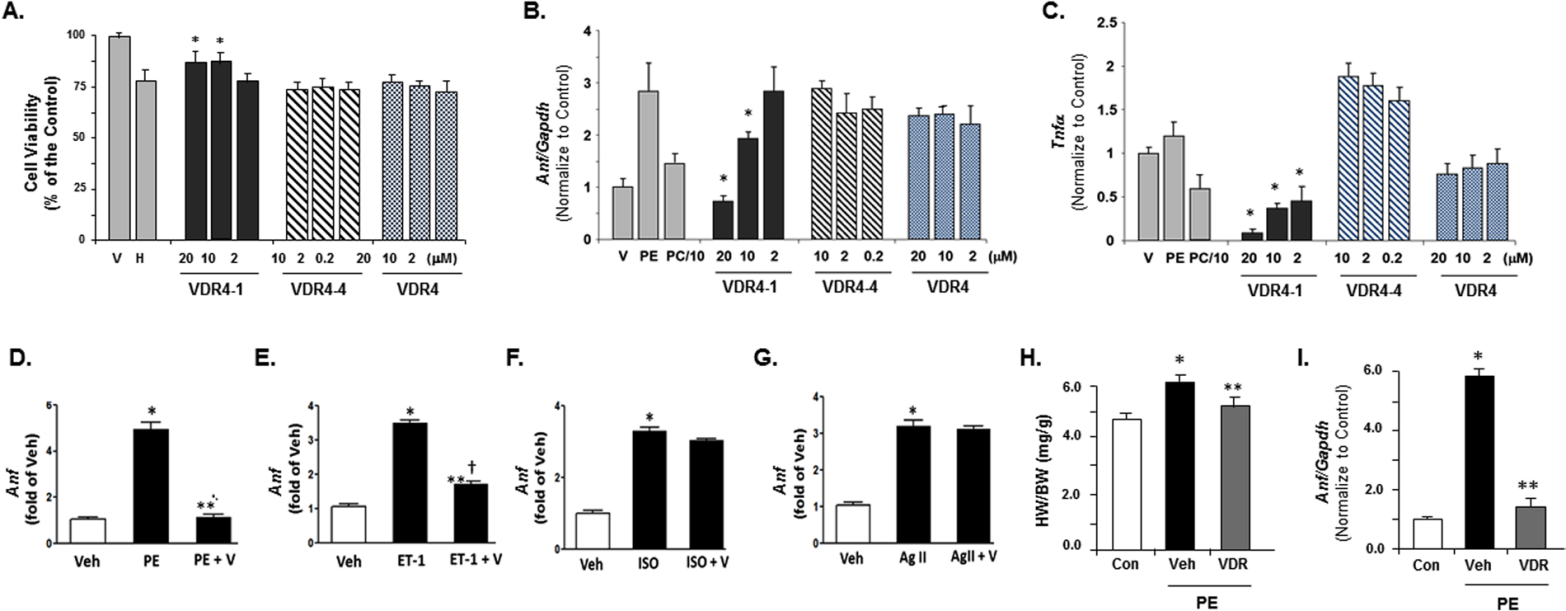
*In vitro* anti-apoptotic and anti-hypertrophic effect of three lead compounds. (**A**) Cell viability assay of three lead compounds on 294 HEK cells. V = Vehicle, H = H_2_O_2_. * =p < 0.05 vs H. N = 4. (**B,C**) Effect of PE on three lead compounds on *Anf* (**B**) and *Tnf-*α (**C**) mRNA expression in ARCM *in vitro*. mRNA level was determined using real-time PCR. *Anf* = atrial natriuretic factor, *Gapdh* = Glyceraldehyde 3-phosphate dehydrogenase, PE = phenylephrine. *p < 0.05 vs PE. N = 4. (**D–G**) Effect of VDR 4-1 on *Anf* mRNA expression to PE (**D**), ET-1 (**E**), ISO (**F**), and AgII (**G**) in ARCM *in vitro*. ET-1 = endothelin-1, ISO = isoproterenol, AgII = angiotensin II. *p < 0.05 vs Veh, **p < 0.05 vs PE or ET-1; N = 4/group. (**H**,**I**) Heart weight/body weight (HW/BW) ratio (**H**) and *Anf* mRNA expression (**I**) in mouse hearts after infusion of PE (30 mg/kg/day) via osmotic pump for 7 days. Con = control, Veh = vehicle, VDR = VDR 4-1 (0.6 μμg/kg). Drugs were injected 3x/week *i.p*. *p < 0.05 vs Con, **p < 0.05 vs Veh; N = 4/group.

Since vitamin D compounds have been suggested to have anti-apoptotic activities^23, 24^, we tested to see if any of these three compounds have protective effects to hydrogen peroxide (H_2_O_2_) induced apoptosis. Induction of apoptosis was achieved with 0.5 mM of H_2_O_2_ resulted in about 30% decreases in cell viability after 24 hours (Fig. 3A). VDR 4-1 at 10μM and 20μM resulted in significant decrease in cell death compared to the vehicle. VDR 4-4 and VDR 4, however, did not show significant cellular protective activities.

Vitamin D deficiency has been associated with development of cardiac hypertrophy and cardiac dysfunction. Our laboratories have previously shown that therapy with vitamin D analog, paricalcitol, can prevent progression of cardiac hypertrophy and attenuate the progression to decompensated heart failure in Dahl salt sensitive rat model^15, 25^. To examine the biological effect of VDR 4-1, we first tested VDR 4-1 for its anti-hypertrophic potential in cultured adult rat cardiomyocytes (ARCMs) exposed to phenylephrine (PE). PE significantly activated atrial natriuretic factor (*Anf*), which is well-established as biochemical evidence of cardiac hypertrophy. We tested different doses of lead agonists, VDR 4-1, VDR 4-4, and VDR 4 to determine the anti-hypertrophic effect in this model. PE resulted in significant activation of *Anf*, suggesting robust biochemical evidence of cardiac hypertrophy in ARCMs (Fig. 3B). There were concentration dependent suppressions of *Anf* level with VDR 4-1, but not with VDR4-4 or VDR4. In addition, we found that VDR 4-1, but not VDR 4-4 or VDR 4, effectively suppressed *Tnf-*α activation induced by PE (Fig. 3C). Thus, we concluded that VDR 4-1 was an effective VDR agonist with significant anti-hypertrophic effect. We used VDR 4-1 for further characterization.

To determine whether these effects are stimuli-dependent, we used three other known hypertrophic stimuli, isoproterenol, endothelin-1 (ET-1) and angiotensin II, to see if VDR 4-1 is able to suppress *Anf* activation induced by these three stimuli. We found that VDR 4-1 significantly suppressed *Anf*-induction by PE and ET-1stimuli (Fig. 3D and E). However, VDR 4-1 did not show significant effect on isoproterenol and angiotensin II induced hypertrophy (Fig. 3F and G). This finding is interesting since renin-angiotensin system (RAS) has shown to be involved in the development of cardiac dysfunction, especially in renal failure^26, 27^. However, our previous study suggested that the anti-hypertrophic effect of vitamin D may be mediated via RAS-independent pathway^25^.

We then evaluated anti-hypertrophic potential of VDR 4-1 in PE-induced cardiac hypertrophy in WT mice *in vivo* ^28, 29^. Infusion of PE over 48 hours resulted in significant increase in heart weight/body weight (HW/BW) ratio and *Anf* activation in left ventricular tissue, which are both hallmark of cardiac hypertrophy (Fig. 3H and I). Administration of VDR 4-1 effectively blocked PE-induced HW/BW ratio as well as PE-induced *Anf* activation.

### Effect of lead VDR 4-1 on serum calcium and parathyroid hormone (PTH)

**Figure 4:**
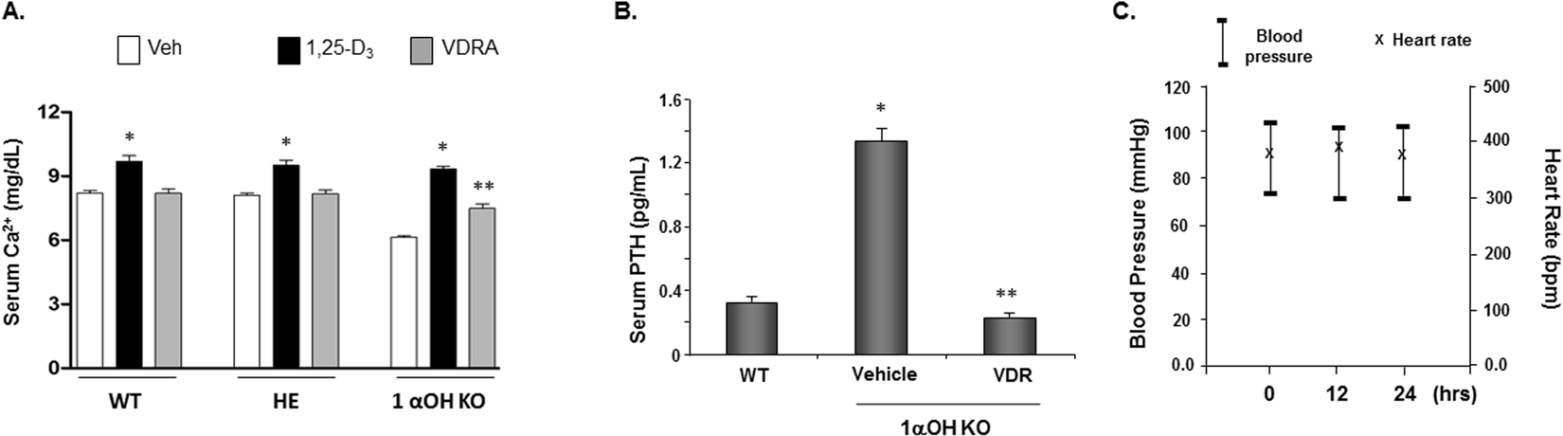
Effect of VDR 4-1 on biochemical and physiological parameters *in vivo*. (**A,B**) Serum Ca^2+^ (**A**) and PTH (**B**) levels after long-term injections (0.6 μg/kg M, W, F for 1 week). 1,25-D_3_ (0.6 μg/kg), VDR = VDR 4-1 (0.6μg/kg) 3 x/week *i.p*. *p < 0.05 vs Veh, **p < 0.05 vs 1,25-D_3_; N = 3/group. WT = wild-type, HE = heterzygote for 1α-HO deletion (1α-HO −/+), 1ααααα-HO KO = homozygote for 1α-HO deletion (1α-HO −/−). (**C**) BP and HR after VDR 4-1 injection (0.6 μg/kg *i.p*.) at different times. N = 4/group.

To identify compounds which are both potent and devoid of the calcification concerns, we compared the calcemic effect of VDR 4-1 against 1,25-D_3_, which is a naturally occurring vitamin D_3_ compound. We first examined the calcemic effect of VDR 4-1 on vitamin D deficient animal. The critical conversion of storage form of 25(OH)Vitamin D_3_ to the hormonally active form, 1,25-D_3_ occurs by 1-α-hydroxylase (1α-OH). The deletion of 1α-hydroxylase in mice (1α-OH KO mice) results in a phenotype mimicking human pseudorickets by 8 weeks of age including hypovitaminosis and secondary hyperparathyroidism (sHPT)^30^. To evaluate the effects of VDR 4-1 in these mice, we gave the equal doses of VDR 4-1 and 1,25-D_3_ at 3 μg/kg three times a week, which is a supraphysiological dose. We found that 1,25-D_3_ resulted in significant increase in serum ionized calcium levels after 3 weeks of administration (Fig. 4A). In contrast, VDR 4-1 demonstrated no significant hypercalcemia compared to the vehicle injected WT group and significantly attenuated the hypocalcemic condition in vitamin D deficient mouse. In addition, we tested the effect of VDR 4-1 on sHPT in 1α-OH KO mice. Administration of VDR 4-1 3x/week injection effectively suppressed sHPT in 1α-OH KO mice (Fig. 4B). These data suggest that VDR 4-1 is a novel non-steroidal vitamin D agonist with effective suppression of sHPT without significant calcemic effect compared to 1,25-D_3_. In addition, blood pressure (BP) and heart rate (HR) did not significantly change with injection at various times (Fig. 4C). From these data, we conclude that our lead molecule VDR 4-1 functions as a VDR agonist; it could exert known biological effects of vitamin D signaling, such as correcting sHPT, without affecting calcium level, BP and HR *in vivo*.

### Effect of VDR 4-1 on the expression of Ca^2+^ transport genes

**Figure 5:**
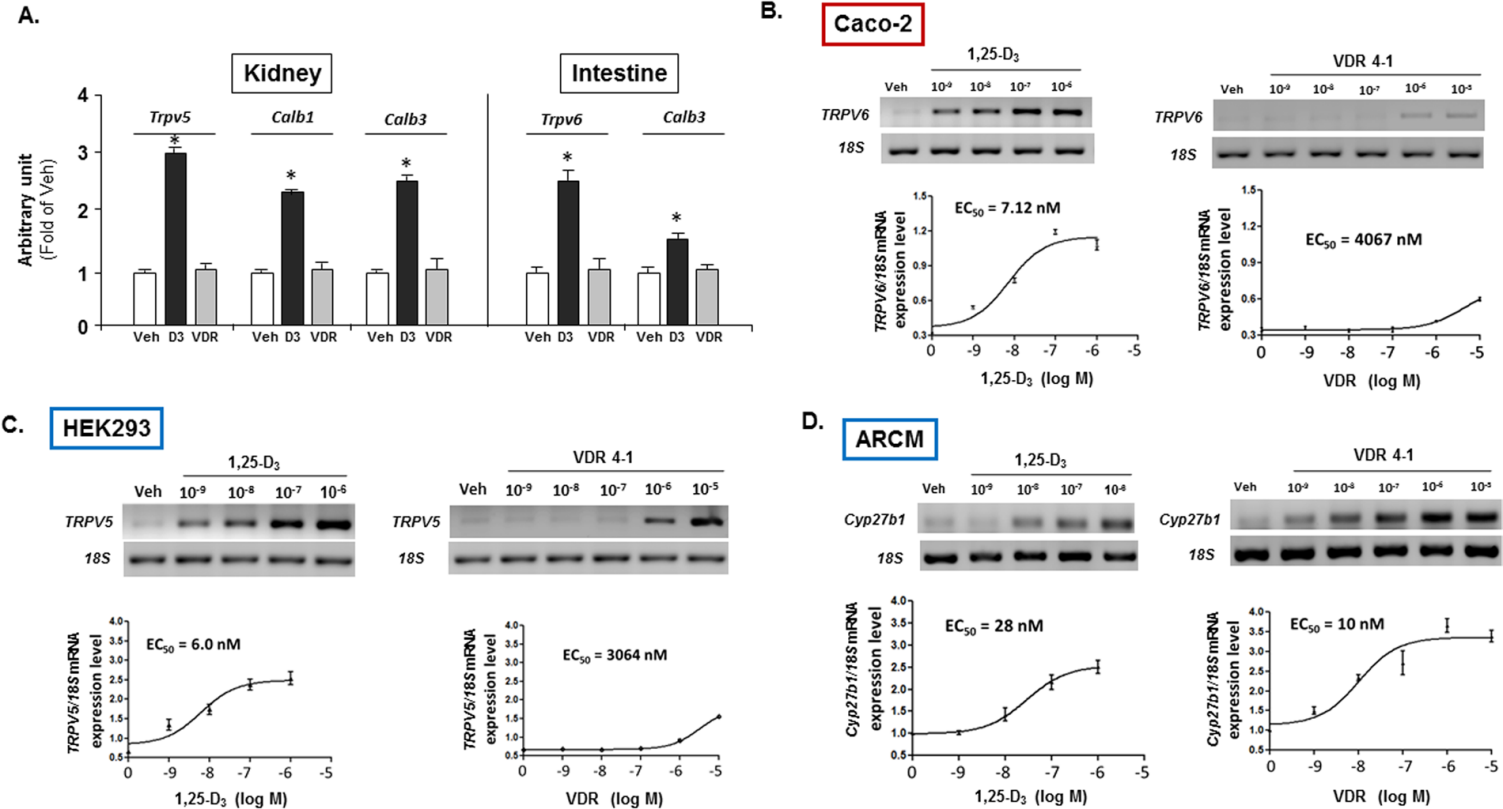
EC_50_ of VDR 4-1 (VDR) compared to 1,25-D_3_ (D3). (**A**) mRNA expression levels of various VDR-responsive genes in the kidney and intestinal tissues after VDR 4-1 administration (0.6 mg/kg). *p < 0.05 vs Veh; N = 3/group. (**B–D**) Representative PCR (upper panels) and EC_50_ graphs (lower panels) of VDR responsive genes in Caco-2 intestinal (**B**) and HEK293 kidney (**C**) and ARCM cells (**D**). Only relevant PCR bands are shown. No alteration was done to the original gel images. *TRPV6*, *TRPV5* and *Cyp27b1* are measured for Caco-2 intestinal (**B**) and HEK293 kidney (**C**) and ARCM cells (**D**), respectively. Calcitriol (left panels) and VDR 4-1 (right panels).

Intestine and kidney tightly regulate body Ca^2+^ hemostasis by passive paracellular and active transcellular Ca^2+^ (re)absorption pathways^31–33^. Renal and intestinal Ca^2+^ transport genes are drastically reduced in VDR-knockout animals^34^. First, to genetically assess the effect of VDR 4-1 on Ca^2+^ transport genes *in vivo*, we gave single, equal injection of supraphysiologic doses (3 μg/kg) of VDR 4-1 and 1,25-D_3_ in the 1α-OHase KO mice. We found that 1,25-D_3_ supplementation significantly increased transient receptor potential cation channel subfamily V member 5 (*Trpv5*), calbindin-D28k (*Calb1*) and calbindin-D9K (*Calb3*) mRNA expression in kidney at 24 hrs compared to vehicle-treated mice (Fig. 5A). Calcitriol also significantly elevated the *Trpv6* and *Calb3* mRNA expression in intestine compared to their vehicle treated littermate. In contrast, our lead VDR 4-1 did not significantly altered the expression of genes encoding Ca^2+^ transport proteins involved in kidney and intestinal transcellular Ca^2+^ (re)absorption compared to vehicle treated groups.

Next, to test the mechanism of non-hypercalcemic properties of VDR 4-1, we used differentiated Caco-2 human intestinal cells, HEK 293 human kidney cells and adult rat adult cardiomyocytes (ARCM) to determine the activation of the VDR-responsive genes to VDR 4-1 in cell specific manner. In Caco-2 intestinal and HEK 293 kidney cells, 1,25-D_3_ showed robust activation of *TRPV6* and *TRPV5* mRNA activation, respectively (Fig. 5B and C). Compared to 1,25-D_3_, the half maximal effective concentration (EC_50_), as measured by *TRPV6* and *TRPV5* mRNA activation, of VDR 4-1 effects were about 600-fold less in CaCo-2 cells and 500-fold less in HEK293 cells. As expected, these results indicated that VDR 4-1 showed lower potency in inducing the expression of the endogenous Ca^2+^ transport genes, whose expression is obligatory for Vitamin D_3_-mediated Ca^2+^ absorption in gut, compared to 1,25-D_3_. To test the effect of VDR 4-1 in heart, we used ARCMs to determine the activation of the VDR-responsive genes to VDR 4-1. EC_50_ for cytochrome P450, family 27, subfamily B, polypeptide 1 (*Cyp27b1*) (gene encoding 1,25-hydroxylase) were 28 and 10 nM for 1,25-D_3_ and VDR 4-1, respectively (Fig. 5D). Compared to 1,25-D_3_, VDR 4-1 was about 3-fold higher potent inducer of *Cyp27b1* in ARCMs. Accordingly, 1,25-D_3_ and VDR 4-1 showed similarly efficacious EC_50_, as measured by VDR mRNA activation, in ARCMs (data not shown).

### Therapeutic effects of VDR 4-1 on cardiac hypertrophy with defective Vitamin D metabolism

**Figure 6:**
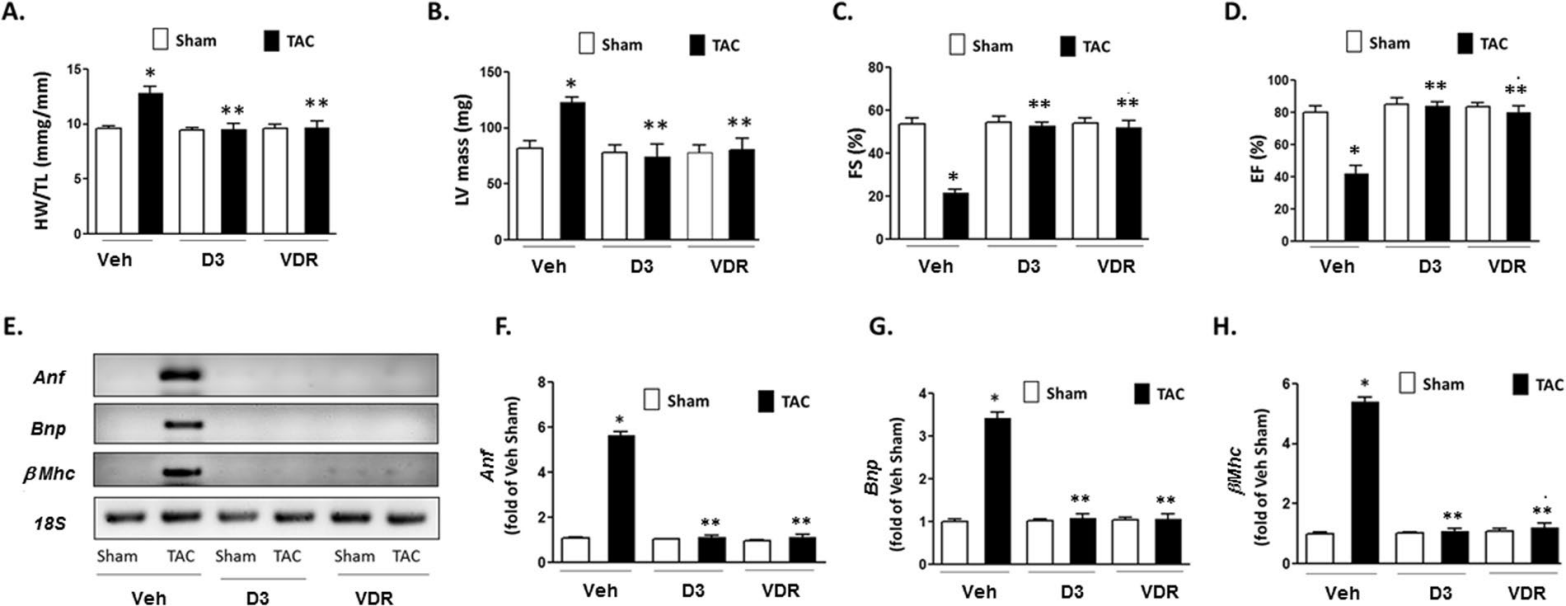
Effect of physiologic dose of VDR 4-1 (0.6 μμg/kg) on vitamin D deficient mice after TAC *in vivo*. (**A–D**) Morphologic and functional assessment of 1α-HO KO mice at 4 weeks after TAC. HW/BW ratio (**A**) LV mass (**B**) Fractional shortening (**C**) and ejection fraction (**D**) are displayed. *p < 0.05 vs Veh-Sham, **p < 0.05 vs Veh-TAC; N = 4–6. (**E**) Representative *Anf*, *Bnp* and μ*Mhc* mRNA expression. Only relevant PCR bands are shown. No alteration was done to the original gel images. *18 S* was used as an internal control for RNA. (**F–H**) Quantification of *Anf* (**F**), *Bnp* (**G**), and μ*Mhc* (**H**) mRNA expression. D3 = 1,25-D_3_ (0.6 μg/kg), VDR = VDR 4-1 (0.6 μg/kg) 3 x/week *i.p*. Total volume injected = 50 μL/animal/injection. *p < 0.05 vs Veh-Sham, **p < 0.05 vs Veh-TAC; N = 4–6.

1α-OH KO mice develop hypocalcemia, sHPT, hypertension, cardiac hypertrophy and impaired cardiac function^30^. To elucidate the effect of VDR 4-1 to pathological conditions with defective vitamin D signaling, we used an established model of cardiac hypertrophy using transverse aortic constriction (TAC). We first used “rescue” vitamin D protocol as described previously in 1α-OH KO mice^35^. TAC resulted in a significant increase in HW/TL and LV mass in mice receiving vehicle for 4 weeks compared to sham operated mice (Fig. 6A and B). In mice treated with VDR 4-1 and 1,25-D_3_ l at physiologic dose (0.6 μg/kg), there were significant attenuations of TAC-induced cardiac hypertrophy compared with their vehicle treated littermates. TAC significantly decreased FS and EF in vehicle treated mice. However, vitamin D replacement with VDR 4-1 and 1,25-D_3_ resulted in significantly attenuation of cardiac dysfunction after TAC (Fig. 6C and D). In addition, both VDR 4-1 and 1,25-D_3_ significantly mitigated TAC-induced increases in *Anf*, *Bnp* and *β-Mhc* mRNA gene expression compared to vehicle treated group (Fig. 6E–H). From these data, we conclude that both 1,25-D_3_ and VDR 4-1 could prevent cardiac hypertrophy with “rescue” concentration of vitamin D *in vivo*.

### Therapeutic effects of VDR 4-1 on cardiac hypertrophy with normal Vitamin D metabolism without calcemic effect

**Figure 7:**
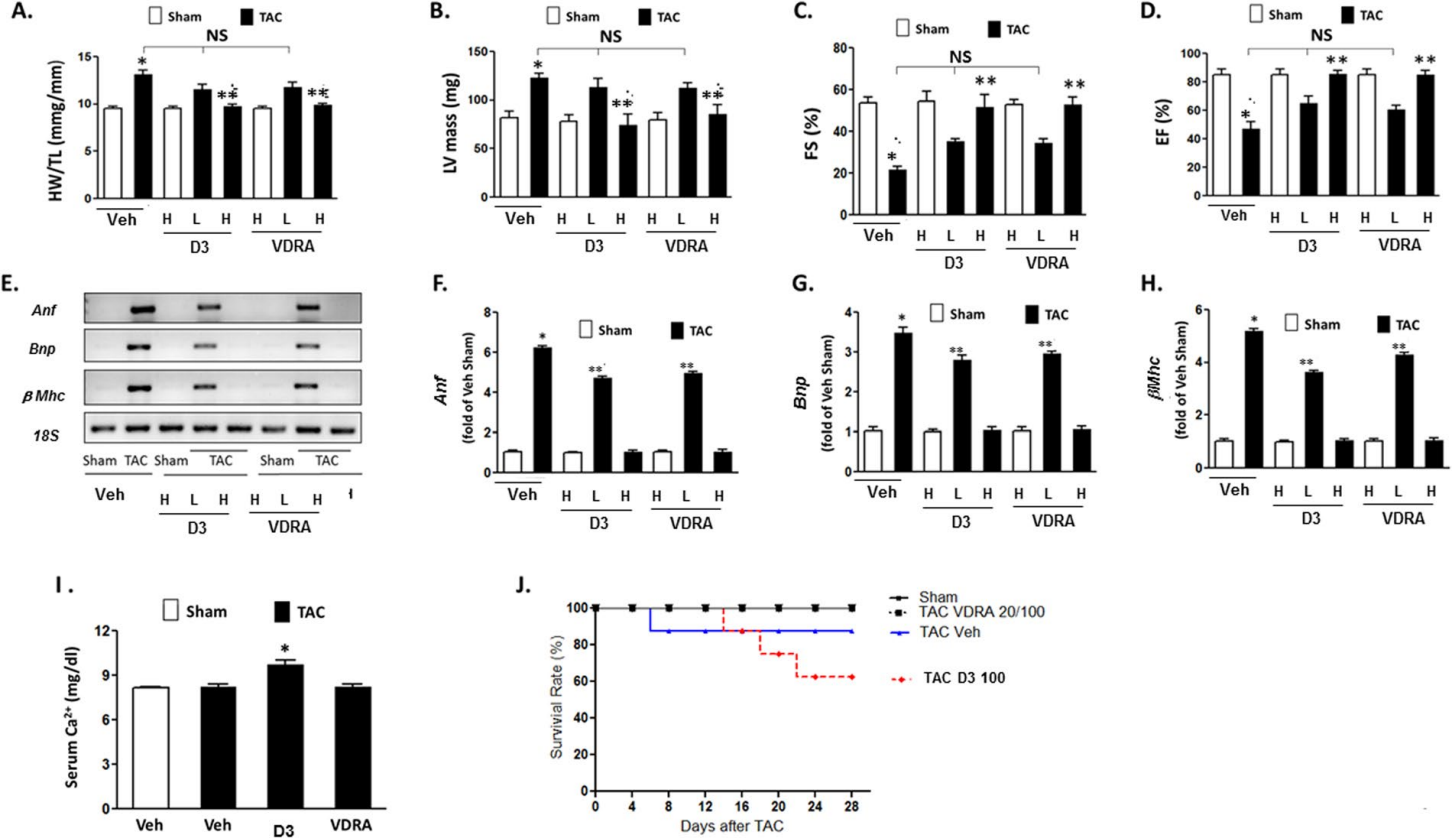
Effect of supraphysiologic dose of VDR 4-1 (3.0 μg/kg) on WT mice with normal vitamin D level mice after TAC *in vivo*. (**A–D**) Morphologic and functional assessment of 1α-HO KO mice at 4 weeks after TAC. HW/BW ratio (**A**), LV mass (**B**), Fractional shortening (**C**) and ejection fraction (**D**) are displayed. *p < 0.05 vs Veh-Sham, **p < 0.05 vs Veh-TAC; N = 4–6. (**E**) Representative *Anf*, *Bnp* and μ*Mhc* mRNA expression. Only relevant PCR bands are shown. No alteration was done to the original gel images. *18 S* was used as an internal control for RNA. (**F–H**) Quantification of *Anf* (**F**), *Bnp* (**G**), and μ*Mhc* (**H**) mRNA expression. *p < 0.05 vs Veh-Sham, **p < 0.05 vs Veh-TAC; N = 4–6. (**I**) Serum Ca^2+^ level after long-term injections D3 = 1,25-D_3_ (3.0 μg/kg), VDR = VDR 4-1 (3.0 μg/kg) 3 x/week *i.p*. Total volume injected = 50 μL/animal/injection. *p < 0.05 vs Veh; N = 4–6. (**J**) Survival curve of mice after supraphysiologic dose of VDR 4-1.

To assess the effect of VDR 4-1 replacement on cardiac hypertrophy with normal vitamin D signaling, we again used TAC-induced cardiac hypertrophy in WT mice. Morphometric analysis demonstrated significant increases in HW/TL ratio and LV mass in WT mice compared with the sham-operated group after 4 weeks of TAC (Fig. 7A and B). Moreover, FS and EF in TAC vehicle treated group were also significantly decreased compared their sham-operated littermate (Fig 7C and D). Physiologic dose (0.6 μg/kg) of VDR 4-1 and 1,25-D_3_ moderately attenuated TAC-induced HW/TL ratio and LV mass with moderate improvement in EF and FS (Fig. 7A–D). However, supraphysiologic dose (3.0 μg/kg) of VDR 4-1 and 1,25-D_3_ significantly attenuated cardiac hypertrophy with significant improvement of cardiac function compared to the TAC vehicle animals. In addition, supraphysiologic doses of both VDR 4-1 and 1,25-D_3_ significantly decreased the expression level of biological markers for cardiac hypertrophy, such as *Anf*, *Bnp* and *β-Mhc* mRNA, compared to their vehicle treated littermate (Fig. 7E–H). Yet, physiologic doses (0.6 μg/kg) failed to significantly attenuate the biochemical markers for cardiac hypertrophy. Importantly, supraphysiologic dose of 1,25-D_3_ resulted in significant increase in serum Ca^2+^ levels and resulted in 30% mortality in these mice after 4 weeks of administration (Fig. 7I and J). In contrast, supraphysiologic dose of VDR 4-1 did not increase serum Ca^2+^ levels or mortality. These data suggest that VDR 4-1 has anti-hypertrophic effect even in normal vitamin D state without causing hypercalcemia, likely due to lack of intestinal and kidney absorption of Ca^2+^, indicating a favorable safety profile.

## Discussion

In this study, we describe a virtual screening approach that leverages the strength of structure-based virtual screens (SBVS) with the computational speed of ligand-based virtual screens (LBVS), enabling search of novel VDR agonists. We used a unique step-wise hypothesis-driven hybrid approach. LBVS-guided screen, which captured important binding-site interacting groups common in known vitamin D agonists, coupled with SBVS in an *ensemble* of receptor structures bound to known agonists of varying size and substitutions to account for receptor flexibility in docking calculations, is a successful strategy to discover novel agonists and reduce the antagonists and false positive hits. For LBVS, a 5-site pharmacophore query was successful at biasing the screen towards known features of VDR recognition and activation. Bypassing LBVS and simply performing single-structure (1DB1) SBVS resulted in few potent antagonists, but no agonist (out of 35 compounds tested), suggesting that structure-based HTD enriched the hits with antagonists. In the next attempt, pharmacophore based LBVS followed by single structure (1DB1) SBVS (37 compounds tested) resulted in weak agonists as well as antagonists, strongly supporting the role of pharmacophore bias in enriching hits with known agonist features, with docking-based filter justifying a role in reducing false positives.

Our more successful approach ‘*ensemble*-SBVS’ involved evaluating pharmacophore hits in multiple VDR conformations. Use of receptor *ensemble* has been reported to be useful strategy for enrichment of hits where induction in receptor conformation as a result of ligand binding is established^36^. Consequently, we were convinced that *ensemble* of three crystal structures was computationally amenable for our virtual screening campaign. This screen resulted in discovery of potent agonists (Suppl. Table S1, compounds 1–5), as evaluated *in vitro* and *in vivo* animal studies. This supports our hypothesis that novel chemical scaffolds with tissue selective VDR modulation properties will be devoid of hypercalcemia. Furthermore, a comparison of the molecular surfaces of Glide predicted binding poses of lead compounds VDR 4, VDR 4-1 and VDR 4-4 to the co-crystallized native ligands in 1DB1, 2HB7 and 3CS6 crystal structures revealed that overall binding surface occupied by our lead compounds overlaps well with the surface of co-crystallized ligands in respective VDR structures (Fig. 2). A detailed discussion of molecular surface comparison and docking poses of lead compounds is included in Supplementary Information.

The key components required for a functional vitamin D-dependent signaling system are found in the heart, including the presence of functional VDR, 1α- hydroxylase and 24-hydroxylase in the ventricular myocardium. Moreover, VDR expression is upregulated with the induction of cardiac hypertrophy^37^, and vitamin D deficiency in cardiomyocytes is associated with abnormalities in contraction, and collagen and renin gene expression^38, 39^. Our laboratory has shown that vitamin D therapy blocks the development of cardiac hypertrophy and progression to decompensated heart failure in Dahl salt sensitive rats fed a high salt diet^15, 25^.

Clinically, children with vitamin D deficiency-rickets suffer from cardiomyopathy^17^, while vitamin D deficiency and hyperparathyroidism are regularly found in patients with severe CHF^18^. Given that the requisite conversion of the storage form of vitamin D to its activated form occurs in the kidneys, the patients with kidney failure are typically vitamin D deficient. In fact, the prevalence of left ventricular hypertrophy and diastolic dysfunction in end stage renal disease is about 80%, and the rate of cardiovascular-related mortality 10–20 times higher in this group than in the general population^40, 41^. Our group has demonstrated that hemodialysis patients treated with paricalcitol have a reduction in LV wall thickness and improved diastolic dysfunction parameters using echocardiography compared with untreated patients^15^. In addition, we previously demonstrated in an observational study that there was improved survival rate in hemodialysis patients treated with paricalcitol, an active vitamin D analog^19^, and this improved survival rate was associated mainly with a decrease in cardiovascular mortality^20^. These data suggest that vitamin D deficiency may be a contributing factor in the pathogenesis of CHF, and that the anti-hypertrophic properties of vitamin D signaling may confer a cardioprotective advantage. However, it is possible that anti-hypertrophic properties (as well as other extra-renal effects) of vitamin D may require higher doses of vitamin D. By devising a VDR agonist compounds that are devoid of significant calcemic effect may be desired in these cases. Thus, based on these findings, our non-steroidal VDR agonist analogs have an exciting potential to be used for various vitamin D deficient states as well as potentially for non-renal vitamin D therapy. Our findings are similar to Ma *et al*. who demonstrated that non-secosteroidal compounds can induce less calcemia *in vivo*, and exhibited improved therapeutic index over the naturally occurring VDR ligand 1,25-D_3_ in an *in vivo* preclinical model of psoriasis^22^.

In conclusion, using a virtual screening protocol, which combined ligand- and structure- based information and biasing our screening towards the chemical features required for receptor activation we identified several non-steroidal VDR agonists and antagonists of human VDR. The biological confirmation, especially the anti-hypertrophic effect of these compounds in the absence of hypercalcemia, is exciting and promising. Further studies are needed to determine whether these compounds eventually could yield novel and clinically viable VDR agonists in humans

## Methods

### Materials

Test compounds (purchased from Chembridge store, www.Hit2Lead.com, San Diego, CA; racemic mixtures if chiral) and Calcitriol (1,25-D_3_; Sigma) were dissolved in 100% DMSO (Sigma) at 1000-fold stock concentration and stored at −20 °C, which were diluted in assay medium immediately prior to use. All methods were performed in accordance with the relevant guidelines and regulations.

### Computational Methods

Methods applied in pharmacophore query generation, Glide docking, ensemble HTD and consensus ranking for compound selection for testing is further included in Supplementary Information. All molecular modeling operations were performed in the Maestro (Schrödinger) and MOE (Chemical Computing Group) modeling packages running on Dell Precision 690 workstation and RHE Linux 5 OS. The chemical libraries employed in the virtual screening primarily included Chembridge, but Asinex, NCI and ZINC databases were also searched during second generation 2D similarity based scaffold expansion. The structures were prepared using Ligprep program (Schrödinger) while Epik^42–44^ (Schrödinger) identified possible ionization and tautomer states at physiological pH (7.0 ± 2.0), along with generating ring conformers and stereoisomers with default settings. The ionization state penalties for each protomer state were calculated and stored for docking score calculation.

### Pharmacophore Elucidation

Pharmacophore hypotheses were generated using 13 highly potent analogs of 1,25-D_3_, energy optimized in the binding pocket of VDR-LBD crystal structure (PDB code: 1DB1). This receptor-aligned set of structures was subjected to pharmacophore elucidation protocol in Phase (Schrödinger) for calculation of sites, followed by identification of common features and hypotheses scoring which take these three components into account: site, vector and alignment scores. A *five-feature* model (DDHHH.30) that survived scoring criteria in Phase^45, 46^ was chosen for databases screening with 1,25-D_3_ being selected as a reference structure for alignment and scoring of this query. This query was used for initial database screening, with at least 4 out of 5 features matching requirement. Similarly, individual ePharmacophore (Schrödinger) were elucidated using crystal structure ligand bound to VDR-LBD (PDB codes: 1DB1, 2HB7, 3CS6) using XP descriptors calculated with Glide^47–49^ docking with ‘score-in-place’ mode. These ePharmacophore were found to compliment key residues interactions offered by Vitamin D binding (Suppl. Fig. S1). Specifically, energetically most significant site H8 (score –1.36) represented C-ring, site H7 (score –0.90) represented D-ring, followed by 2 donors (D; scores –0.70) representing hydroxyl groups at 1 and 3 positions in A-ring of 1,25-D_3_, and involved in hydrogen bonding with Ser237 and Ser278. The hydrophobic features H8 and H7 represented fused C-D ring in 1,25-D_3_ and stabilized by sticky residues Leu230, Ala231, Leu233, Val234, Ile268, Ile271, Leu313, Val418. The hydrophobic features H8 and H7 represented fused C-D ring in 1,25-D_3_ and stabilized by sticky residues Leu230, Ala231, Leu233, Val234, Ile268, Ile271, Leu313, Val418.

### Virtual Screening

Hits from pharmacophore screen (fitness score ≥1.5) were subjected to structure-based docking evaluations using X-ray crystal structures of human VDR-LBD in complex with: 1,25-D_3_ (PDB code: 1DB1), 2α-(3-hydroxy-1-propyl)-1,25-D_3_ (PDB code: 2HB7) and the super agonist AMCR277B (a cyclic side-chain analog of 1,25-D_3_; PDB code: 3CS6), nominally resolved at 1.5–1.8 Å range. The structures were prepared using the protein preparation wizard utility in Maestro, in which ions and water molecules were deleted and all hydrogen atoms were added. The ionization states of bound ligands and charged side chains of the protein amino acids were calculated followed by optimization of hydrogen bond network and constrained energy minimization of only hydrogen atoms, up to RMSD of 0.3 Å. For each PDB conformation, the receptor grids were generated for active sites with native ligand as the grid center. The side chain hydroxyl groups of critical amino acids (S237, S274 and Y143) in binding pocket participating in hydrogen bonding were treated flexibly during docking simulation. Preliminary docking was performed in standard precision (SP) scoring function, followed by more rigorous evaluation of hits with SP scores ≥−8.0 kcal/mol using extra precision (XP) settings, with consideration to ionization state penalty in docking score calculation.

The top hits from XP docking with docking scores better than −8.0 kcal/mol were considered for post-docking analysis. The top-scoring pose of each molecule was assigned a unique rank based on the docking score in the respective VDR conformation. Similar ranking was assigned to these molecules based on pharmacophore scores. This ranking metric, which consisted of three rankings based on docking scores in 3 VDR conformations and one for fitness score, was taken into account for consensus versus individual receptor ranking using MOE database utility. Final selection of molecules was mostly based on visual inspection of docked poses and compounds availability for purchase.

### High Throughput Binding Assays

The GeneBLAzer^®^VDR -UAS-bla HEK 293 T cell-based assay (Invitrogen, Cat#K1700) was used to screen 181 compounds for VDR agonists. Upon binding of a VDR agonist to the targeting construct (GAL4-DBD/VDR-LBD) fusion protein engineered in a HEK293T cell line containing 7XUAS-bla, a transcriptional cascade is initiated that produces β-lactamase (*BLA*). In the presence of the *BLA* LiveBLAzer™ substrate, cells expressing *BLA* will fluoresce blue (460 nm), while those not expressing *BLA* will fluoresce green (530 nm); higher 460/530 ratio indicating the activation of VDR, whereas in presence of 1,25-D_3_ (potent natural agonist) diminishing of this ratio indicates an antagonist behavior of the test molecule. The agonist and antagonist abilities of *in silico*-selected compounds were determined in six-point concentration profile and compared with vehicle.

The assay was run in triplicate following the manufacture’s instruction. Briefly, 2 × 10^4^ cells in 32 µl assay medium were added to each well of 384-well black-wall assay plate (Corning Incorporated, Cat# 3712). After addition of 8 µl/well compound diluted in assay medium, the cells were incubated in a humidified 37 °C/5% CO_2_ incubator for 5 hours. The cell-free wells (containing 32 µl assay medium only) and unstimulated cell wells were treated in parallel with the same concentration of DMSO in assay medium, which were used as background controls and blank controls, respectively. Calcitriol was used as a positive control. To each well, 8 µl of LiverBLAzer-FRET B/G substrate mixture were added. The plate was covered with plate sealer and incubated at room temperature for 2 hours in the dark. Fluorescence emission was measured on SpectraMax plate reader at 460 nm and 530 nm with excitation set to 409 nm.After subtraction of background, average value at 460 nm (blue color) was divided by average value at 530 nm (green color) to obtain the 460/530 ratio.

### Animal care, animal surgery and hemodynamic measurement

Experiments were conducted using 8–10-week old male mice and their littermates. 1α-OHase + /− and 1α-OHase −/− mice were produced from 1α-OHase + /−^30^. All mice, breeders and offspring were housed at the Animal Research Facility at Beth Israel Deaconess Medical Center (BIDMC). TAC was performed in 8–10 week old male mice as described previously^50^. Cardiac function was analyzed using echocardiography (for baseline cardiac function) and left ventricular (LV) pressure-volume loop measurement (after TAC) as described previously^15, 25, 50^. Euthanasia was performed by CO_2_ via a gas cylinder. All experimental procedures were approved by the Institutional Animal Care and Use Committee of Beth Israel Deaconess Medical Center (BIDMC). All animal use and care were in accordance with the BIDMC animal management program, which is accredited by the American Association of the Accreditation of Laboratory Animal Care (AAALAC) and meets National Institutes of Health standards as set forth in the “Guide for the Care and Use of Laboratory Animals” (DHHS publication No. (NIH) 85-23 JR Rev., 1985).

### Adult Cardiomyocyte Culture and Cytotoxic and Apoptotic Stimulation

Primary cultures of cardiomyocytes from 6-week-old Sprague-Dawley rats were prepared as described^51, 52^. To induce hypertrophy, cardiomyocytes were treated with phenylephrine (PE, 100 μM) or endotheline-1 (ET-1, 10 nM) for 24 hours.

### Plasma Brain Natriuretic Peptide

Tail vein blood was collected at baseline, 3, and 6 weeks (at sacrifice) after initiation of a HS diet. Plasma levels of *BNP* were measured using *BNP* ELISA kit (Assaypro, St. Charles, MO) according to the manufacturer instructions.

### Real-time PCR, RT-PCR and Histological analyses

PCR analyses were performed as described previously^15, 25, 50^. Cardiac histological analyses were performed as described previously^15, 25, 50^.

### Statistical Analysis

Data were expressed as means ± SEM. Comparison between and within groups were conducted with unpaired Student *t* tests and repeated measures ANOVA, respectively. Probability (*p*) values of <0.05 were considered statistically significant.

## Electronic supplementary material

Supplementary Information
