# Supplementary material for: Identification of Novel Non-secosteroidal Vitamin D Receptor Agonists with Potent Cardioprotective Effects and ﻿﻿devoid of Hypercalcemia
Source: Sci Rep. 2017 Aug 16;7:8427. doi: 10.1038/s41598-017-08670-y (PMC5559458; doi:10.1038/s41598-017-08670-y)

Supplementary Info

Identification of Novel Non-secosteroidal Vitamin D Receptor Agonists with Potent Cardioprotective Effects without Inducing Hypercalcemia

Santosh A. Khedkar[1,5], Mohamed A. Samad[2], Sangita Choudhury[2], Ji Yoo Lee[2], Dongsheng Zhang[3], Ravi I. Thadhani[4], S. Ananth Karumanchi[3], Alan C. Rigby[1,*,†], Peter M. Kang[2,*]

[1] Division of Molecular & Vascular Medicine, [2] Cardiovascular Division, and [3] Division of Nephrology, Beth Israel Deaconess Medical Center, Boston, MA; [4] Renal Division, Massachusetts General Hospital, Boston, MA; [5] Current address: ChemBio Discovery Solutions (www.ChemBioDiscovery.com).

<u>Supplementary Methods:</u>

Structure Based Virtual Screening Using *Ensemble* of Receptor Conformations:

Use of receptor *ensemble* has been reported to be useful strategy for enrichment of hits where induction in receptor conformation as a result of ligand binding is established. Rueda *et al.* previously reported that the protein conformations co-crystallized with the largest ligands were those providing the highest individual AUC values (29). Consequently, we selected three co-crystal structures based on the size of co-crystallized ligands: the ligand in 2HB7 structure bears a hydroxypropyl substitution at C2 of A-ring (C-atom between two hydroxyl groups), whereas 3CS6 ligand structure has its C17-side chain (on D-ring) cyclized, with reference to 1,25-D ligand structure in 1DB1, making it apparent that these modifications in 1,25-D structure would have induced the observed conformational changes in VDR-LBD structures, as evident from the

maximum RMS deviation of 5.6 Å in protein heavy atoms. We were convinced that *ensemble* of three crystal structures was computationally amenable for VS as well as provide local inductions in VDR-LBD pocket due to structural changes in different parts of 1,25-D (Suppl. Fig. S1). Consequently, this screening set selected based on three Glide XP score-based consensus rankings for three respective crystal structures and fourth ranking for pharmacophore fitness, identified five potent agonists (Suppl. Table S3; compounds 1–5), and five weak antagonists (Suppl. Table S1, compounds 11-15) out of 51 compounds tested in transcriptional binding assay. Compound 4 (Suppl. Table S3; referred as VDR 4 hereafter) was selected as lead candidate for further chemical space expansion.

Among the optimized lead series analogs (Suppl. Table S4), VDR 4, VDR 4-1 and VDR 4-4 were further evaluated in *in vitro* and *in vivo* animal studies. A comparison of the molecular surfaces of Glide predicted binding poses of lead compounds VDR 4, VDR 4-1 and VDR 4-4 to the co-crystallized native ligands in 1DB1, 2HB7 and 3CS6 crystal structures revealed that overall binding surface of lead compounds overlap well with the surface of co-crystallized ligand in respective VDR structure. However, it is observed that binding of lead compounds (Fig. 1 and Suppl. Fig. S2) in 1DB1 and 3CS6 structures have a progressive penetration towards A-ring sub-pocket, with VDR 4-1 and VDR 4-4 being pushed-down, possibly due to methoxy and methyl substitution on D-ring, respectively. The acyclic side chains on bicyclic BC-ring of VDR 4-4 in 1DB1 and 3CS6 structure do not run parallel to native vitamin D aliphatic side chain, unlike in cases of rest of the poses with an exception of VDR 4 binding to 2HB7 structure, in which VDR 4 binds after rotation along the longitudinal axis (~180°) so that cyclic (D-ring) and acyclic ester substitution on C-ring try to switch their places in an attempt to push the compound down longitudinally to occupy the A-ring sub-pocket similar to the hydroxypropyl chain on A-ring of

ligand in 2HB7. Overall, a mutual presence of hydrophobic substitutions of appropriate length and size at R1/R4 may be essential to mimic the shape (physical fitting) and electrostatics (affinity) to interact with H11/H12 helix and exhibit agonist activity, as docking predicted R1 and/or R4 substitutions to occupy the binding pocket accessed by lipid side chain of 1,25-D. Long/bulky side chain (R1) is necessary for agonist activity when D-ring substitutions are smaller or absent (e.g., VDR 4-3), a balance between groups/bulk at D-ring and side chain is important; this part of molecules heads towards H11-12. Bulk at both R1 and R4 (VDR 4-27 and VDR 4-31) may not leave sufficient room in H11-12 region to fit or align in appropriate manner to stabilize the interactions. A noteworthy observation, which could be a demonstrating example of consistent activity within this series, is that B and C rings in scaffold VDR 4 series of molecules align on C- and D-rings of vitamin D seco-steroidal skeleton, consistent with the most favorable features for binding (scores –1.36 and –0.90, respectively) as per ePharmacophore energetics.

Binding Pocket Interactions of Lead Compounds 4, 4-1, and 4-4:

In a comparative analysis, a pattern in the surface area (SA) of VDR agonists and their transcriptional response was observed: compound **4** (SA, 391 Å^2; agonist) < compound **4-45** (400 Å^2; antagonist) < 3CS6-ligand (417 Å^2; agonist) = 1DB1-ligand (419 Å^2; agonist) = compound **4-22** (417 Å^2; agonist) < compound **4-12** (457 Å^2; agonist) < 2HB7-ligand (471 Å^2; agonist). It may be concluded from this observation that increasing the SA of bioactive conformation of ligand seems to improve agonist potency of these ligands. A close look at the molecular interactions of lead compounds revealed that A-rings in compounds **4**, **4-1** and **4-4** are stabilized by pi-pi interactions with Tyr143 in the Glide docking predicted poses in this study. In

addition, D-ring in compound **4-4** also pi-stacks with Trp286, which may be attributed to smaller methyl substitution at *para* position in compound **4-4** versus relatively larger methoxy substitution at *meta* position in compound **4-1**. In general, similar binding orientation of acyclic side chain in lead compounds with the C-17 side chain in 1,25-D analogs opens great opportunities to reposition this chemical space to improve potency and ADMET properties. A sequence of structure-based designs followed by binding energy evaluations of modified analogs was undertaken using Embrace and Glide programs that shed light on this, suggesting potential favorable modification on this scaffold: removal of D-ring or substitution with smaller rings, in compound **4**, replacement of acyclic ester side chain in scaffold **4** with most favorable side chains of known 1,25-D analogs for improving hydrogen bonding network with Ser237, Ser278, Arg274 or Tyr143 by substituting A-ring with hydrogen bond donor/acceptor groups along with suitably long linkers. There is also a room for improving hydrogen bonding interactions of acyclic side chain with H305 and H397.

It is worth noting that chemical scaffold of compound **4** series has two chiral carbon centers (*), one on each of the fused rings (C-D) linking A and D rings, however, the compounds used in this study were purchased as racemic mixtures and not chirally pure enantiomers. Though there is a strong possibility of one specific stereochemical preference over the other for binding of these compounds to VDR-LBD, it remains difficult to draw any conclusions based on the results in this study. Within this limited SAR data, we observed that a variety of groups at R1 position could be accommodated. According to Glide predicted binding conformations of this series of compounds, R1-bearing long side-chain corresponds to the binding position of aliphatic side chain found on D-ring of 1,25-D, and can favorably accommodate long or bulky (such as phenyl) groups when substitutions on D-ring are smaller or absent, as in essence this D-ring and

R1 side chain compete for the same space in binding pocket and influence the overall binding of this series of compounds. For instance, compound **4** has long side chain at R1 and no substitutions on D-ring (R2=R3=R4= H) so that the side chain takes preference in long channel of hydrophobic pocket. It was observed from structure-activity relationship and Glide predicted binding modes of this set of analogs tested in transcription assay that R1 and/or R4 substitutions seem to imitate the interactions of 1,25-D lipid side chain that may in turn be essential to stabilize the helix-12 (H12) in appropriate conformation; the H12 contains a critical, ligand modulated interface for the interaction with coactivator proteins. Therefore, mutual presence of hydrophobic substitutions of appropriate length and size at R1/R4 may be important for agonist activity.

We were aware of the fact that both agonist and antagonist bind tightly to LBD, however their ability to activate VDR is a function of their structural features that induce conformational changes after binding. It was revealed that the orientation of individual feature in the binding site (location) and its relative importance (energetic contribution) to ligand binding was in line with the requirements of binding interactions of known agonists.

Supplementary Tables

Table S1: Screening hits exhibiting VDR antagonist activity in experimental transcription binding assay (530/460 ratios) at various compound concentrations.

**Compd.#**	**Chemical Structure**	**530/460 ratio at**				
		**0.05**	**0.5**	**5**	**50**	**100**
1	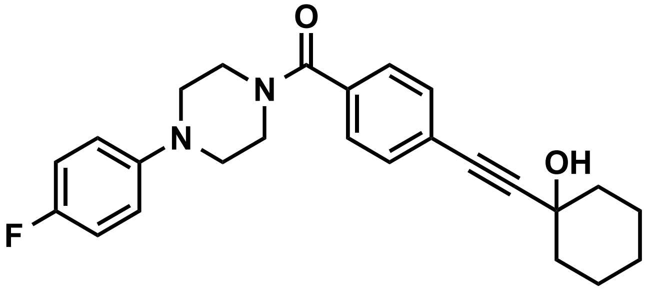	NT	**2.9**	**2.9**	**0.1**	NT
2	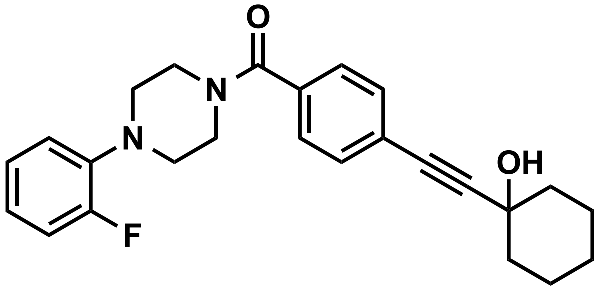	NT	**3.2**	**3.0**	**0.1**	NT
3	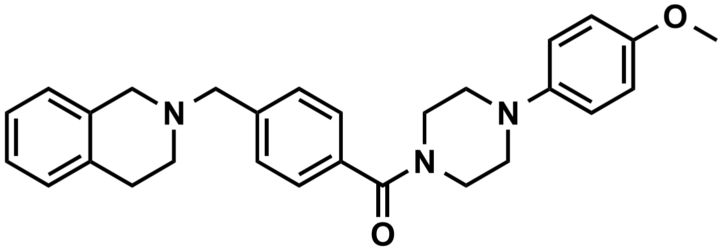	NT	**3.3**	**2.6**	**0.1**	NT
4	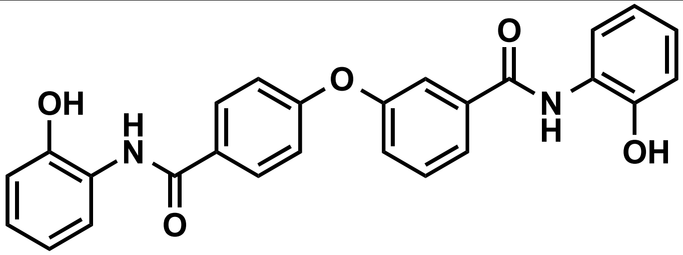	NT	**3.3**	**2.6**	**0.3**	NT

5	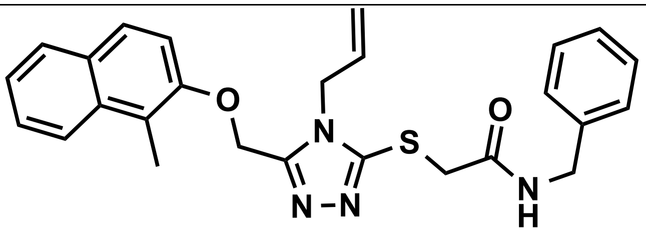	NT	**3.0**	**2.3**	**0.1**	NT
6	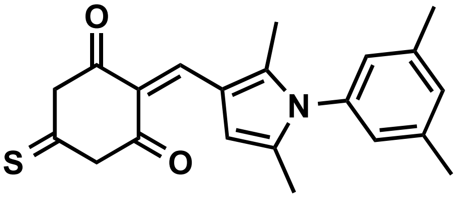	**3.5**	**2.6**	**1.0**	**0.1**	NT
7	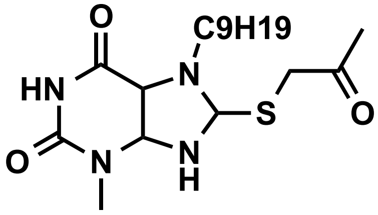	4.4	4.4	4.5	0.4	NT
8	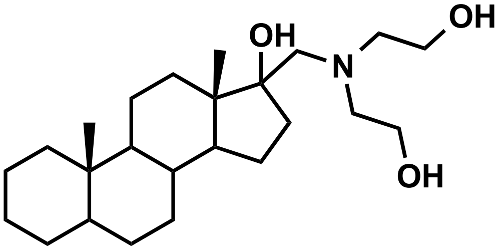	2.9	3.6	3.2	0.3	NT
8	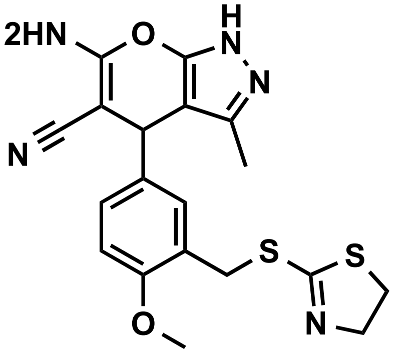	**4.0**	**3.7**	**3.6**	**0.2**	NT
10	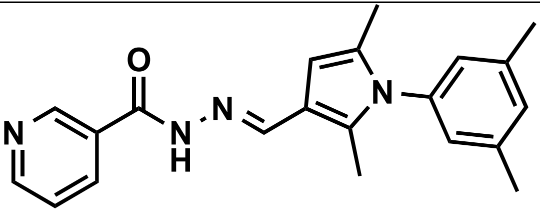	**3.0**	**3.0**	**2.3**	**0.4**	NT

11	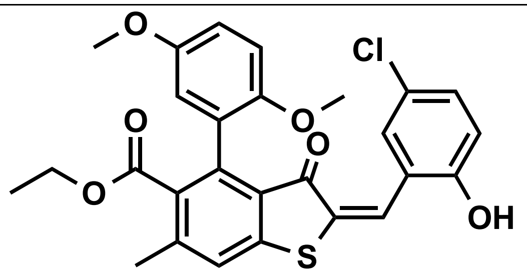	NT	NT	2.0	1.8	1.5
12	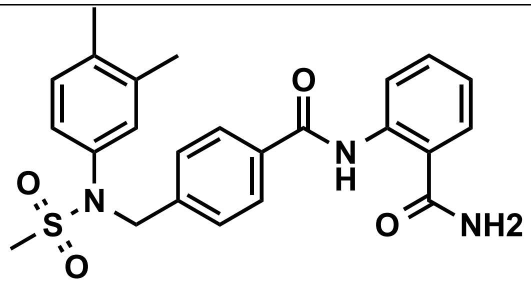	NT	NT	2.3	1.0	0.8
13	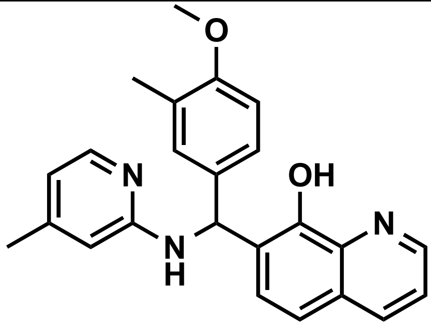	NT	NT	2.8	2.0	1.4
14	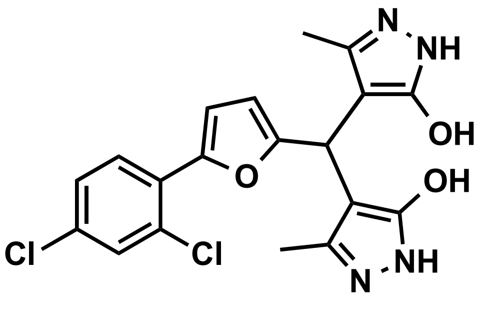	NT	NT	2.3	0.6	*tox
15	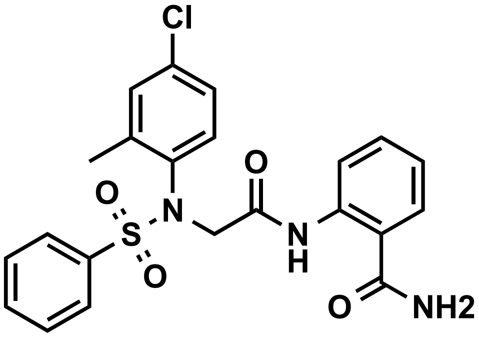	NT	NT	2.3	0.7	0.5

NT, not tested

Table S2: Screening hits exhibiting VDR agonist activity in experimental transcription binding assay (460/530 ratios) at 500 and 1000 uM compounds concentrations.

Compd.#	Chemical Structure	460/530 ratio at	
		500uM	1000uM
1	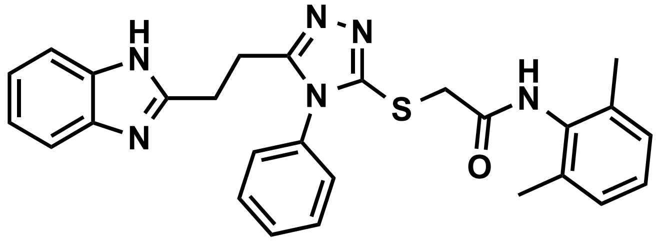	1.5	1.8
2≠	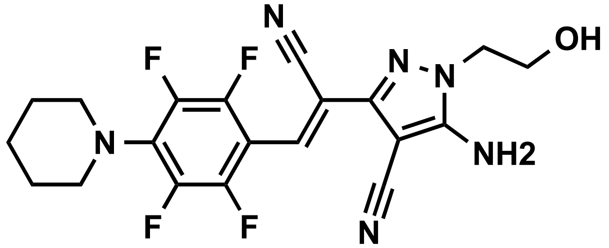	1.1	1.1
3	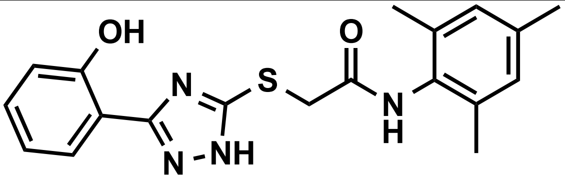	**2.5**	**5.5**
4	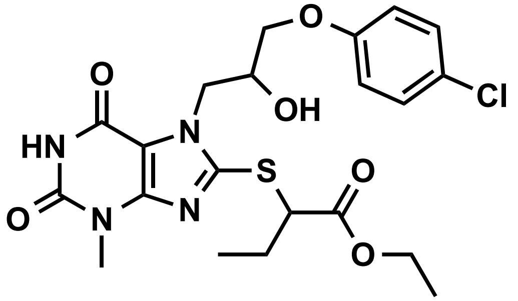	1.4	2.7
5	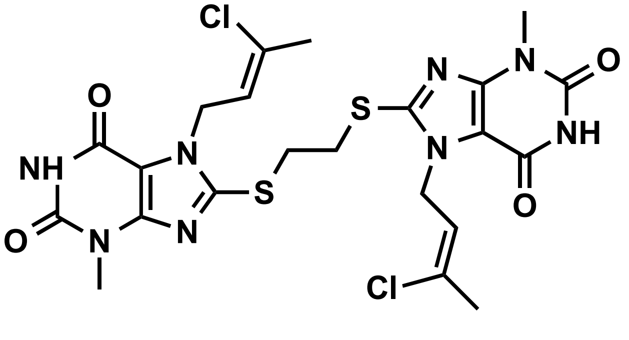	**2.1**	**2.5**

6	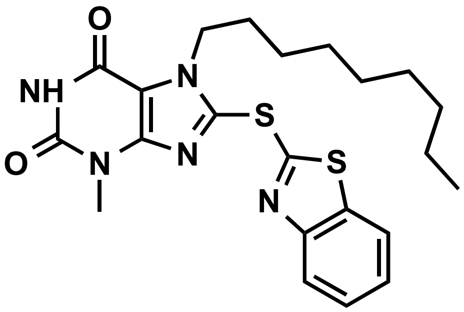	1.5	2.1
7	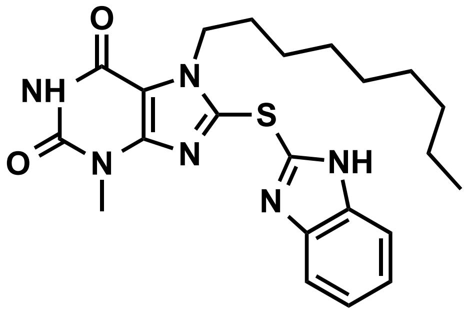	**2.8**	**7.0**
8	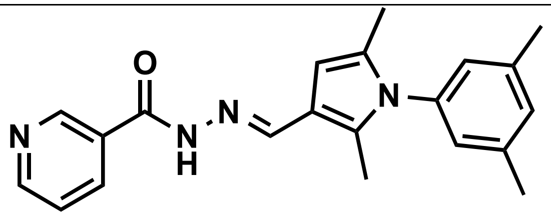	0.9	1.5

≠ Compound 2 showed agonist action at higher concentration, but exhibited as antagonist at very low concentrations (not shown)

Table S3: First generation of compounds (*ensemble HTD hypothesis*) exhibiting agonist activity (460/530 ratios) in VDR transcriptional assay at various compound concentrations.

Compd.#	Chemical Structure	5µM	50µM	100µM	200µM
**1** (VDR 1)	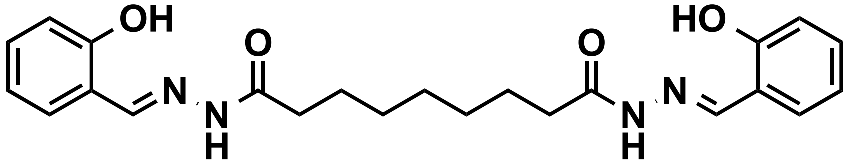	0.8	1.0	1.2	2.1
**2** (VDR 2)	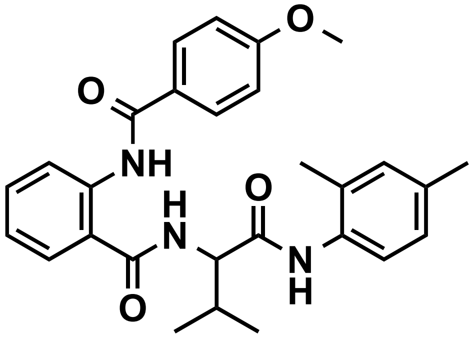	0.6	0.8	0.9	2.5
**3** (VDR 3)	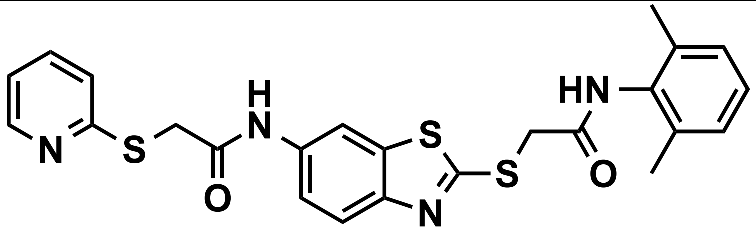	0.8	0.8	1.9	2.4
**4** (VDR 4)	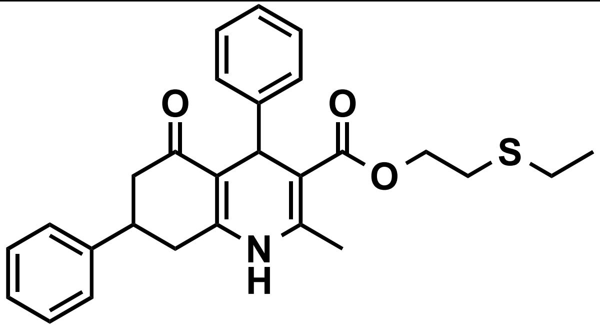	0.6	1.2	6.2	8.5
**5** (VDR 5)	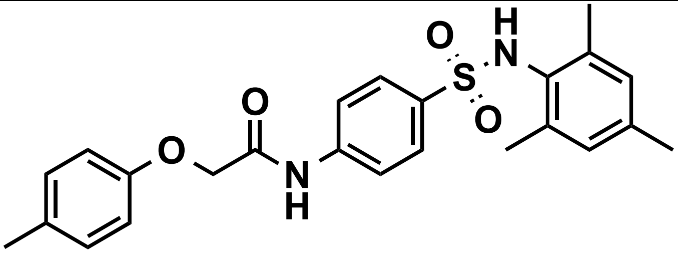	0.6	0.9	1.9	4.5

Table S4: Chemical space expansion efforts and related VDR binding data for lead compound VDR **4**.

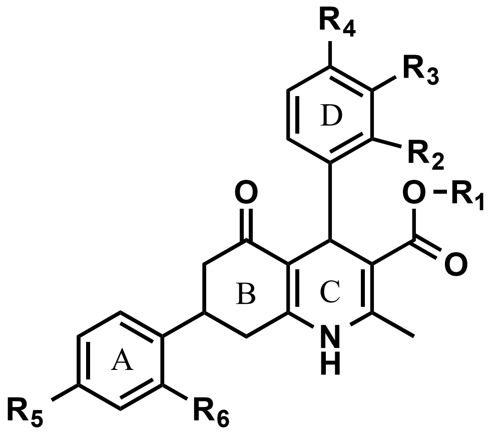

VDR #	@5µM[€]	@50µM[€]	R_1	R_2	R_3	R_4	R_5	R_6
**4**	++	++	CH2-CH2-S-C2H5	-	-	-	-	-
**4-1**	++	+++	CH2-CH2-S-C2H5	-	OCH3	-	Cl	-
**4-2**	+++	+++	CH2-CH2-S-C2H5	OCH3	-	-	Cl	-
**4-3**	+++	+++	CH3	-	OC2H5	OC2H5	-	-
**4-4**	+++	+++	CH2-CH2-O-CH3	**-**	-	CH3	Cl	-
**4-5**	+++	+++	CH2-CH2-O-Ph	-	-	Cl	OCH3	-
**4-6**	+++	+++	CH2-CH2-S-C2H5	-	Br	-	Cl	-
**4-7**	+++	+++	CH2-CH2-O-Ph	-	-	Cl	Cl	-
**4-8**	++	+++	CH2-CH2-S-C2H5	F	-	-	Cl	-
**4-9**	++	+++	CH2-CH2-O-Ph	-	-	-	OCH3	-
**4-10**	++	+++	CH2-CH2-O-Ph	D-ring is 4-pyridine			-	-
**4-11**	++	+++	CH2-CH2-O-Ph	-	-	F	-	-
**4-12**	++	++	CH2-CH2-O-C2H5	OCH3	-	-	-	-
**4-13**	+++	+	CH2-CH2-O-CH3	-	-	Br	Cl	-
**4-14**	+++	+	CH2-CH2-CH3	-	-	Cl	Cl	-

VDR #	@5µM[€]	@50µM[€]	R_1	R_2	R_3	R_4	R_5	R_6
**4-15**	+++	+	CH2-CH2-O-Ph	-	-	OH	OCH3	-
**4-16**	++	+	CH2-CH2-S-C2H5	-	Cl	-	Cl	-
**4-17**	++	+	CH2-CH3	-	-	F	Cl	-
**4-18**	++	+	CH2-CH2-S-C2H5	-	-	F	-	-
**4-19**	++	+	CH2-CH2-O-C2H5	-	-	Br	-	-
**4-20**	++	+	CH2-CH2-S-C2H5	-	-	Cl	OCH3	-
**4-21**	++	+	CH3	-	-	OCH3	-	-
**4-22**	++	+	CH2-CH3	-	-	OC2H5	Cl	-
**4-23**	++	+	CH3	-	-	C2H5	-	-
**4-24**	++	+	CH2-CH2-CH3	-	-	C2H5	-	-
**4-25**	++	+	CH2-CH3	-	OCH3	OC3H7	-	
**4-26**	+	+++	CH2-CH2-S-C2H5	OCH3	-	-	OCH3	-
**4-27**	+	++	CH2-CH2-O-Ph	-	-	C2H5	OCH3	-
**4-28**	+	++	CH2-CH2-S-C2H5	F	-	-	-	OCH3
**4-29**	+	++	CH2-CH2-S-C2H5	F	-	-	OCH3	-
**4-30**	+	++	CH2-CH2-O-C2H5	-	-	Cl	Cl	-
**4-31**	+	++	CH2-CH2-O-Ph	-	-	C2H5	-	-
**4-32**	+	+	CH2-CH2-O-Ph	-	OH	OCH3	-	-
**4-33**	+	+	CH2-CH2-S-C2H5	-	OC2H5	OH	OCH3	-
**4-34**	+	+	CH2-CH2-O-C2H5	**-**	-	F	Cl	-
**4-35**	+	+	CH2-CH2-O-C2H5	-	-	C2H5		-
**4-36**	+	+	CH2-CH2-O-CH3	-	-	C2H5	-	-

VDR #	@5µM€	@50µM€	R_1	R_2	R_3	R_4	R_5	R_6
**4-37**	+	+	CH3	-	-	OC3H7	OCH3	-
**4-38**	+	+	CH2-CH2-S-C2H5	-	-	OCH3	OCH3	-
**4-39**	+	+	CH2-CH3	-	-	OCH3	-	-
**4-40**	+	+	CH3	-	-	OC2H5	OCH3	-
**4-41**	+	+	CH2-CH2-O-CH3	-	-	CH3	OCH3	-
**4-42**	Antagonist		CH2-CH3	-	OH	-	OCH3	-
**4-43**	Antagonist		CH2-CH3	-	-	OH	OCH3	-

All compounds tested as racemic mixture (resulting from chiral carbons in fused B and C rings); €Qualitative presentation of relative VDR transcriptional activities at 5 and 50 µM compound concentrations in cell based GeneBLAzer assay: agonist activities for compounds **4-1** to **4-41** and antagonist activities for compounds **4-42** and **4-43**: more + signs indicate greater relative activity; Compounds **4-1** to **4-12** exhibit better agonist activities both at 5 and 50µM relative to parent compound **4-0**, compounds **4-13** to **4-25** exhibit better agonist activities at 5µM but not at 50µM, compounds **4-26** to **4-31** show better agonist activities at 50 µM but not at 5 µM, compounds **4-32** to **4-41** exhibit lowered agonist activities relative to parent compound **4-0**, at both concentrations.

Table S5: Chemical space expansion efforts and related VDR response for lead compound VDR **5**.

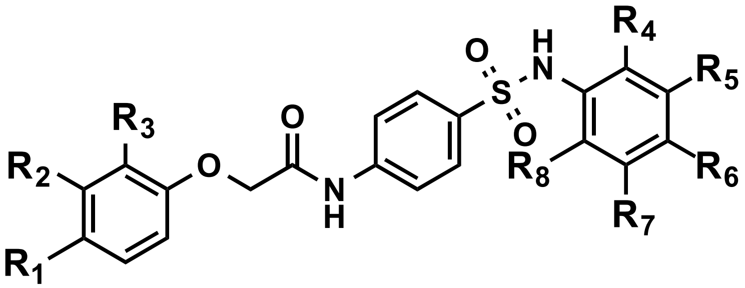

Compd.#	Action	R_1	R_2	R_3	R_4	R_5	R_6	R_7	R_8
5-1	Agonist	CH3	-	-	CH3	-	CH3	-	CH3
5-2	Agonist	Cl	-	-	CH3	-	CH3	-	CH3
5-3	Agonist	-	CH3	-	CH3	-	CH3	-	CH3
5-4	Antagonist	C2H5	-	-	-	CH3	-	CH3	-
5-5	Antagonist	Cl	-	Cl	CH3	-	CH3	-	CH3
5-6	Antagonist	-	CH3	-	-	-	CH3	-	CH3
5-7	Antagonist	-	-	CH3	-	-	OCH3	-	-

A.

Compound **4-1**
(Chembridge/ hit2lead ID: 5711992):

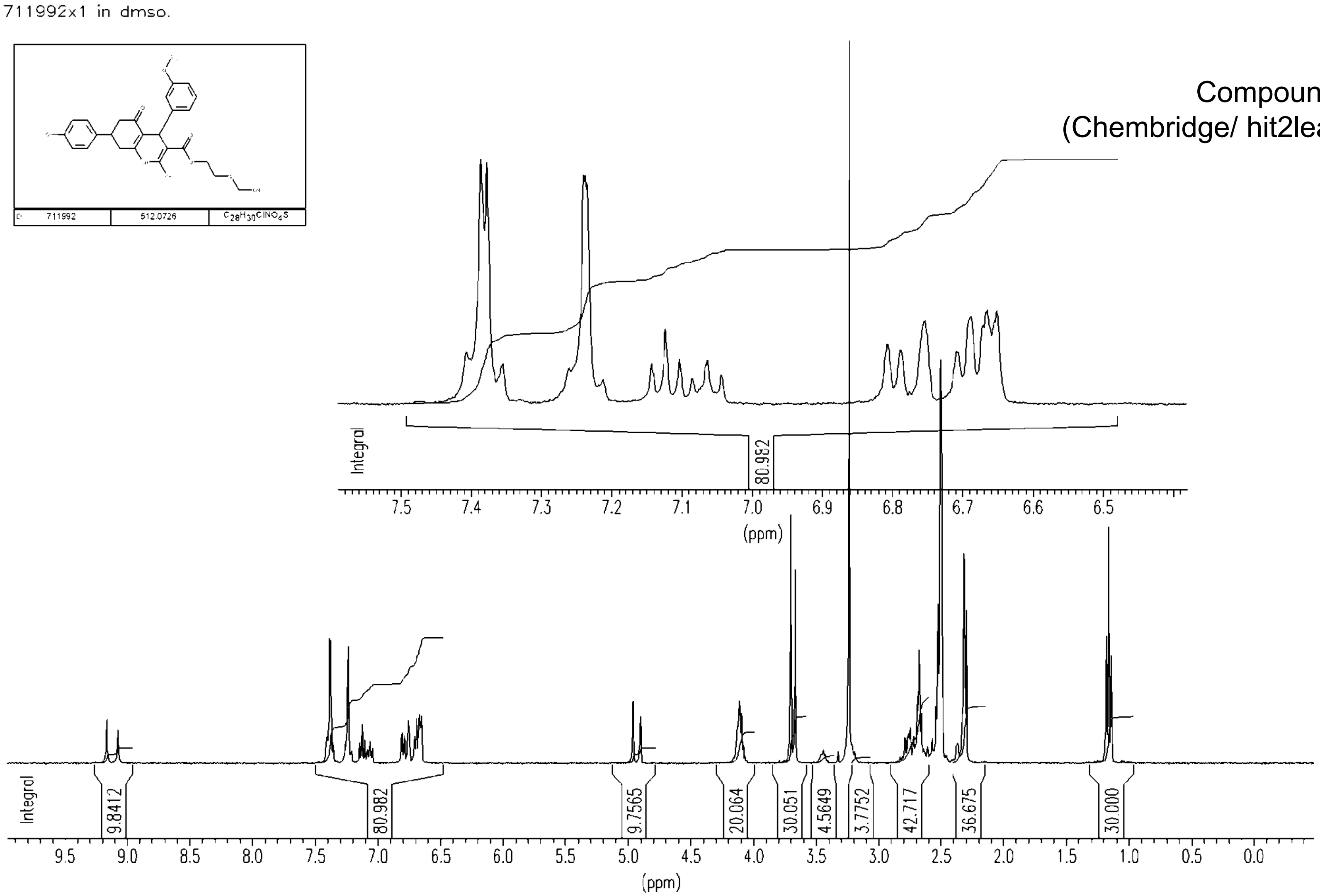

Figure S1: Nuclear Magnetic Resonance spectra of the lead compounds (A) VDR **4-1** (Chembridge/ hit2lead ID: 5711992), **(B)** VDR **4** (Chembridge/ hit2lead ID: 5714991), and (C) VDR **4-4** (Chembridge/ hit2lead ID: 5718040).

B.

Compound VDR **4**
(Chembridge/ hit2lead ID: 5714991):

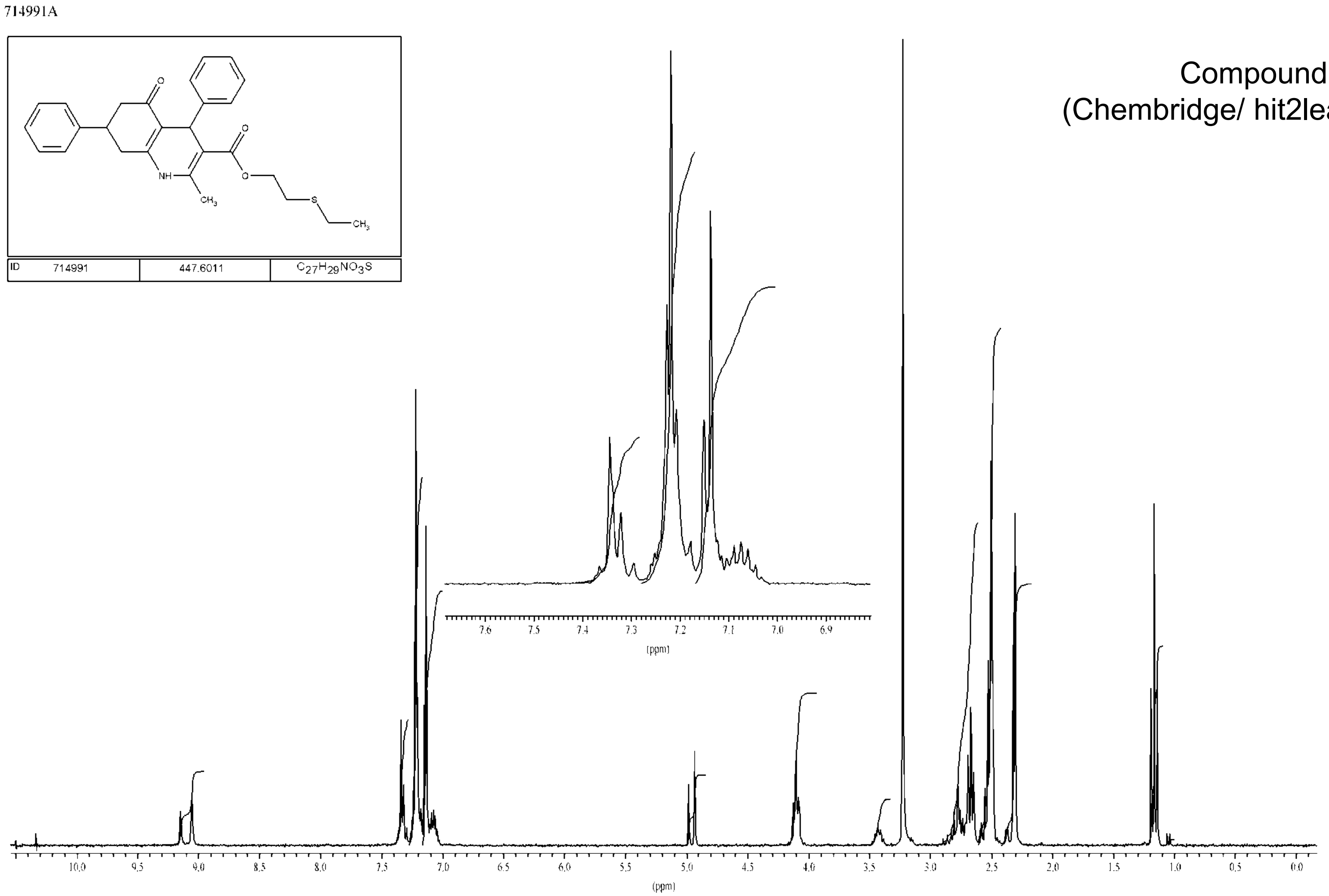

C.

Compound VDR **4-4**
(Chembridge/ hit2lead ID: 5718040):

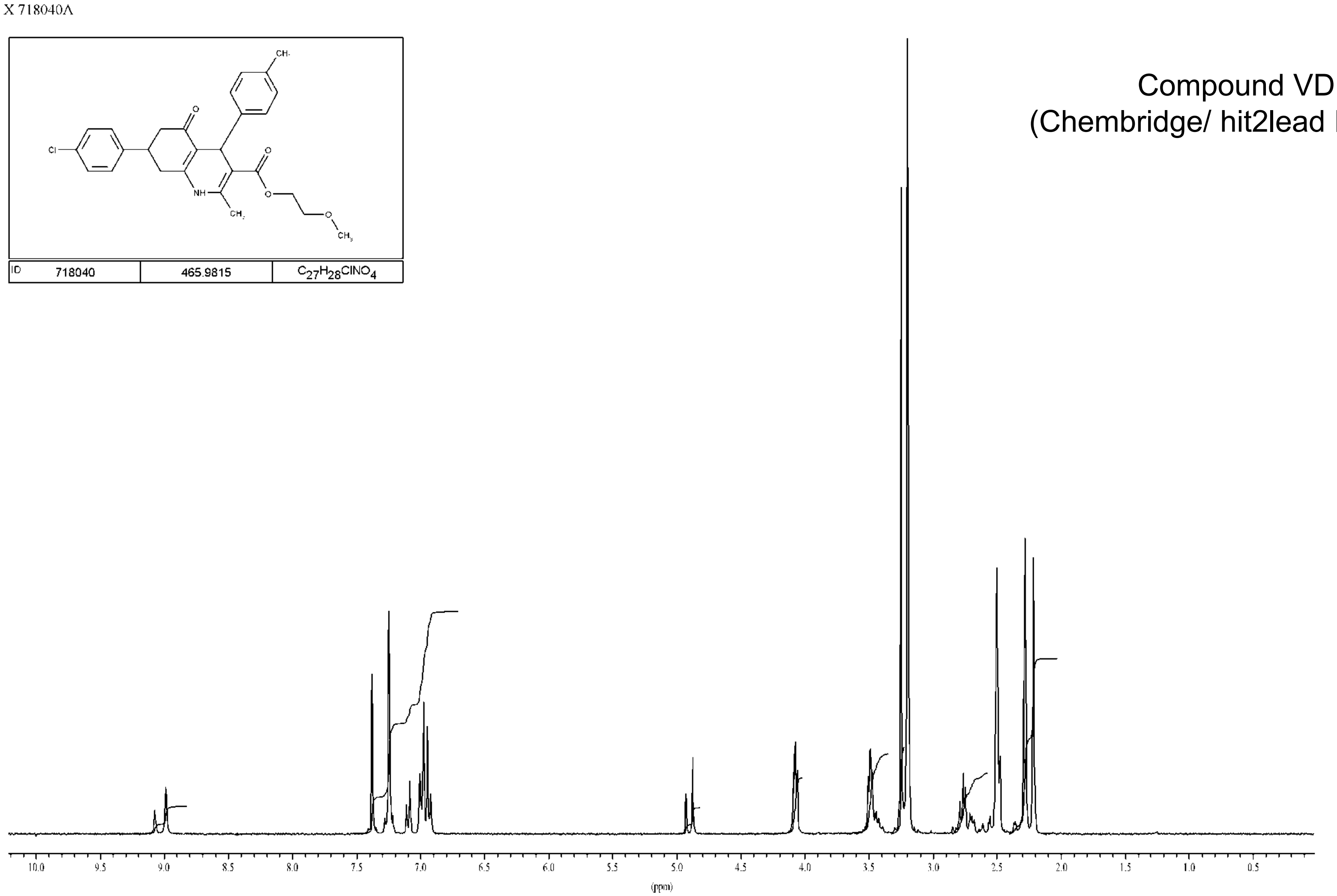

Supplement: Supplementary file 1 — Supplementary Information [file 41598_2017_8670_MOESM1_ESM.pdf]
